# A systematic review on the role of trust in the water governance literature

**DOI:** 10.1016/j.wroa.2022.100147

**Published:** 2022-06-27

**Authors:** Remko Voogd, Peter M. Rudberg, Jasper R. de Vries, Raoul Beunen, Aileen Aseron Espiritu, Nadine Methner, Rasmus Kløcker Larsen, Gunn Elin Fedreheim, Sander Goes, Elizabeth Kruger

**Affiliations:** a Strategic communication Group, Wageningen University and Research, PO Box 8130 , 6700 EW, Wageningen, The Netherlands; b Stockholm Environmental Institute (SEI), Linnégatan 87D, 115 23 Stockholm, Sweden; c Department of Environmental Sciences, Open University, PO Box 2960, 6401 DL Heerlen, The Netherlands; d Barents Institute at The Arctic University of Norway, Faculty of Humanities, Social Sciences and Education, Rådhusgata 8, N-9900 Kirkenes, Norway; e African Climate & Development Initiative, University of Cape Town, 13 Library Road, 7701, Rondebosch, South Africa; f GeoViable, 14940 Cordoba, Spain

**Keywords:** Trust in water governance, Conceptualization of trust, Stakeholders, Sustainable collaboration, Empirical foundations, Systematic literature review

## Abstract

Trust is generally considered to play a key enabling role in water governance. Despite this notion, there have been no systematic assessments examining the way in which the literature on water governance engages with ‘trust’. Our article fills this gap by providing an overview of the way in which this literature has engaged with trust as a conceptual lens, analytical device and empirical phenomenon. Through an explorative systematic literature review of *N* = 200, mainly peer-reviewed journal articles, our findings reveal that the knowledge base on the role of trust in water governance is fragmented, poorly conceptualized, and contextually dispersed. We also observe that the role of trust is often understudied, especially in the context of the global south and with regard to ethnic minorities and indigenous people as the subjects of trust. We recommend that future research should build on solid empirical evidence, diversify its foci, go beyond an instrumental approach to trust and rely on clear and transparent conceptualizations that acknowledge the context-specific and dynamic nature of trust relationships. The results of this review should serve to better systemize future research and to further the understanding on the role(s) of trust in varying contexts and related to different water governance issues.

## Highlights

• We provide the first systematic review on the role of trust in water governance.
• The knowledge base on the role of trust in water governance is fragmented.
• Trust is poorly conceptualized and operationalized in water governance research.
• Trust is understudied in the context of the global south and ethnic minorities.
• Studies on trust in water governance are often instrumentally motivated.

## Graphical abstract

**Figure:**
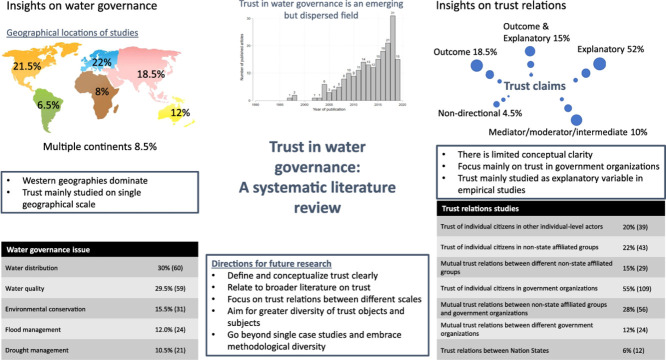

## 1 Introduction

Recent years have witnessed growing academic attention to the role of trust in water governance (e.g. De Vries et al., 2017; Lubell, 2007; Onencan et al., 2018; Wheeler et al., 2017). Trust is deemed important because water governance often requires collaboration and coordination between a wide range of public and private stakeholders. These stakeholders are often bound by different geographical and functional jurisdictions (Lubell and Lippert, 2011), they may have different (conflicting) interests concerning various aspects of water governance (such as water safety, quality, supply, and ecology) (Edelenbos and van Meerkerk, 2015), and they often develop diverse perspectives on problems and their consequent solutions (Benson and Jordan, 2010). Unsustainable land use and increasing scarcity intensifies competition for water while climate change simultaneously requires that additional efforts are made to provide protection against drought and the occurrence of water-related hazards (Woodhouse and Muller, 2017).

In such complex circumstances, the development of mutual trust between stakeholders is supposed to be necessary to facilitate shared understanding and concerted action (e.g. Ansell and Gash, 2007; van Meerkerk and Edelenbos, 2014). Trust between stakeholders is a means to deal with the complexity and uncertainty of interactions as the need to continuously monitor and enforce future actions will be less imminent under conditions of mutual trust (Lubell, 2007; Onencan et al., 2018). Therefore, it is assumed that trust facilitates long term collaboration (Stern and Baird, 2015) and fosters cooperation and compliance by both the wider public and stakeholders directly involved with public policies and environmental management practices (Lafuente et al., 2018; Stern, 2008).

Statements about the essential role of trust for sustainable collaboration also abound in the literature on water governance practices (e.g. Hamm et al., 2013; Leahy and Anderson, 2008; Rogers and Hall, 2003). Nevertheless, it is not known to what extent such statements rely on shared conceptualizations of trust and are underpinned by solid empirical evidence. The knowledge base on trust in water governance seems fragmented (Pahl-Wostl, 2015) and it remains unclear what the possibilities are for valid systematic comparisons of empirical findings on the role of trust. For example, there is limited understanding of how studies on the role of trust in water governance are influenced by *variations* that may exist across different water governance sub-issues (e.g. flood protection, drought management, water quality, environmental protection), geographical contexts, and scales. In addition, attempts to evaluate the knowledge base of articles and to systematically compare their findings may also be hindered by different conceptualizations of the concept of trust itself in water governance studies (Davenport et al., 2007; Lijeblad et al., 2009; Pahl-Wostl, 2015; Stern and Coleman, 2015). Finally, for the comparability of research findings, we believe it is also of value to get an overview of the research approaches and methods that are employed.

To address these knowledge gaps, this article provides – to our knowledge - the first systematic overview of how the water governance literature engages with ‘trust’ as a conceptual lens, an analytical device, and empirical phenomenon, and it reveals whether engagement with trust varies along the lines of some of the structural features of the water governance field (such as sub-issues, geography and scales). To provide this overview, we conducted an explorative systematic literature review, adapted for our needs in the context of an emerging research field in the social sciences (e.g. Petticrew and Roberts, 2006; Torraco, 2005).

The next section of this article (Section 2) theoretically justifies the criteria on the basis of which we evaluate the way in which trust is studied in the field of water governance. Subsequently, we describe how those theoretical considerations informed our research design, our method, literature selection, and our data extraction protocol (Section 3). The centrepiece of our article presents the results of the systematic review (Section 4). The review concludes with a discussion and lines for future research (Sections 5 & 6).

## 2 Aspects of the literature that we review and justification of our analytical criteria

### 2.1 Boundaries within the field: sub-issues, geography and scales

We understand water governance as “the range of political, social, economic and administrative systems that are in place to develop and manage water resources, and the delivery of water services, at different levels of society” (Rogers and Hall, 2003, p. 7). As such, we consider interactions between stakeholders that shape and are part of these systems as important elements of water governance. Although all articles that we review fit under the generic label of being studies on water governance, several studies more particularly focus on specific sub-issues such as flood protection, managing the consequences of drought, water-quality management, and environmental protection. As these various issues all have their own distinct structural elements and most likely involve different sets of actors, it is not guaranteed that the extent to which trust appears, and the way in which it functions, is similar when breaking down the research field in different thematic sub-areas. Thus, assessing how studies on the role of trust in water governance practices are distributed and differ among various sub-issues of water governance is a first important aspect incorporated in our review.

Geographic locations constitute a second type of structural element in the literature in the sense that the role of trust in water governance issues may more often be studied in some locations than others. Moreover, the actual way in which trust is studied may also differ substantially between different locations and cultures. The distinction between developed versus developing countries could be especially relevant in this regard as several challenges of water governance are most acute in developing countries while the conditions for trust-building are at the same time more challenging (Araral and Wang, 2013; Pahl-Wostl, 2015). In addition to location-specific distinctions, there is also a need to distinguish between water governance issues at different geographical scales. The role of trust in establishing sustainable water governance practices may be different at the local scale than at larger-scale (regional, national, cross-boundary) settings where the levels of complexity and uncertainty are different, often requiring decision-making at a larger (or multi-level) scale to achieve satisfactory outcomes (Pahl-Wostl, 2015; Woodhouse and Muller, 2017). Therefore, we deem it important to investigate to what extent studies on the role of trust in water governance vary with regard to geographic locations and scales.

### 2.2 Studying trust: Conceptual underpinning and operationalization

Trust has widely been studied in various social and management sciences (e.g. Hamm, 2017; Nielsen, 2011; Uslaner, 2018), from different perspectives (e.g. Fulmer and Gelfand, 2012; Stern and Coleman, 2015) and with different conceptualizations (Lubell, 2007; Rousseau et al., 1998). Despite this diversity, most applied studies that conceptualize trust share the idea that trust is basically a psychological state of a truster (subject of trust) comprising positive expectations (or negative in case of distrust) that a trustee (object of trust) has certain competences and the goodwill to successfully perform an action on which the truster runs the risk of facing negative consequences (Rousseau et al., 1998; Siegrist et al., 2000). In its most basic form, a trust relation has been summarized by Hardin (2002, p. 9) as “A trusts B concerning matters X”. More recently, an extended formulation designates that “a truster A trusts (judges the trustworthiness of) a trustee B with regard to some behavior X in context Y at time t” (Bauer, 2019, p. 2). Following this latter definition, trust is not only a relational attitude of the truster (A) towards the actions of the trustee (B), but is, at its basic level, context-specific and dynamic. To theoretically ground empirical studies on trust, and to make them better comparable, means that complete assessments of trust relationships should provide a clear conceptualization in which they ideally acknowledge the issue-specific nature of trust (which acknowledges that A trusts B to perform a specific task, but may be less trusting regarding another task (Lewicki et al., 2006)) while simultaneously taking into account that trusters may adapt their expectations over time (Bauer and Freitag, 2018). However, to what extent applied studies provide clear definitions of trust and whether conceptual or empirical descriptions of trust incorporate complete accounts of trust relationships (including elements A to Y) is nebulous. As such, gaining an overview to what extent, and in which way, trust is conceptualized emerges as a first conceptual issue for our review. In addition, investigating to what extent trust is incorporated in the research questions or problem statements of articles provides further insights into the extent to which the concept of trust is fully, and coherently, incorporated in the research designs of articles.

Being specific about who are the subjects (A) who are trusting, and the objects (B) who are trusted is another key point in understanding trust relations. When it comes to the subject of trust (the trusters), it is generally agreed that trust has its basis in individuals or groups of individuals (Bauer, 2019). In this perspective, collective-level units such as organizations or political institutions are not themselves capable of trusting each other. Only the collectively held trust orientation of the group members of such organizations or institutions make it possible to speak about collective-level trust relationships such as inter-organizational trust (Zaheer et al., 1998). Others, however, argue that the subject of trust may also take the form of a group (Stern and Coleman, 2015). The latter approach highlights that collectively defined trust orientations of collective-level actors may become forces in themselves which are able to shape the individual-level trust orientations of ingroup members (Elias and Scotson, 1994).

When it comes to the object of trust (the trustee), trusters may first place trust in other individuals. In its dyadic form, such individual-level trust relations may vary from trust in close relatives to trust in more distant actors (such as individual politicians or other officeholders). Such dyadic trust relations are often spoken of as instances of interpersonal trust (Simpson, 2007) (a conceptualization we follow in this paper, in contrast to authors who use interpersonal trust to designate an individual's general tendency to trust others (Johnson-George and Swap, 1982)). Besides trust in individuals, trusters commonly also direct trust to collective-level entities such as social groups, private companies and government organizations (institutional trust) (Zaheer et al., 1998). Finally, trust in abstract objects - such as formal rules, norms, principles, and (scientific) knowledge – is sometimes classified as an additional object category of trust (e.g. Cockerill et al., 2004; Dalton, 2004).

Given this diversity, several actors – both at the individual and collective level – may be the actual subjects and/or objects of trust in real-world trust relationships. In the water governance context, various individual actors (such as citizens, farmers, ecologists, water managers, or particular officeholders) as well as collective actors (such as water management organizations, NGOs, and all kinds of government branches) can be either subject or object of trust. However, to what extent studies on trust in water governance actually consider different subjects and objects of trust relevant for their specific inquiry, and whether this matters for the findings on trust, is currently not known. Another priority for our review should therefore be to trace whether the literature on trust in water governance clearly specifies between subjects and objects of trust and examine the relationships that appear in real-world trust relationships. Furthermore, we deem it important to know whether the role of trust differs for different subject-object combinations.

Finally, several articles on trust theory from the social and management sciences break down the concept of trust into different subtypes of trust. A commonly adopted perspective – that already takes into account who are the subjects and objects of trust - distinguishes between the general tendency to trust others (appearing under various labels such as ‘social trust’ or ‘interpersonal trust’) and institutional trust (trust based upon expectations that organizations/institutions will act according to the ideals of impartiality, fairness and efficiency) (Seifert, 2018; Zaheer et al., 1998). Additionally, scholars also distinguish between subtypes of trust based on characteristics of the subject of trust and the processes leading to trust (its antecedent). This results in a commonly accepted distinction among; a) trust as stemming from relatively stable psychological attributes of individual trusters, b) trust as stemming from cognitively based calculative processes, and c) trust as based upon affinities and socially embedded properties of relationships between people (Rousseau et al., 1998; Stern and Coleman, 2015). As analytical frameworks that break down the concept of trust to its component parts are arguably more fruitful in explaining trust relationships in real-world contexts than more basic understandings of trust (Stern and Coleman, 2015), identifying to what extent trust is conceptualized regarding its component parts is a third conceptual issue that we address in our review on the role of trust.

### 2.3 Trust in water governance empirically studied: Approaches and methods

To establish a coherent understanding of how trust is empirically studied in the domain of water governance issues, we believe it is also of value to get an overview of the diverse research approaches and methods that have so far been deployed. In line with the fragmented nature of the knowledge base in water governance issues, individual case studies abound in the field (Pahl-Wostl, 2015). But as appropriate research designs need to capture as much of the complexity of water governance processes as possible, scholars have advocated a shift towards comparative case-study approaches and a focus on methodological pluralism (Cook and Bakker, 2012; Pahl-Wostl and Lebel, 2011). We agree that exploratory analyses comprising a large number of cases and in-depth case studies can complement each other (e.g. Pahl-Wostl, 2015). Therefore, we investigate the existing diversity in the research approaches and (data collection) methods in the set of articles that empirically assess the role of trust. As trust may both be a facilitator as well as an outcome of water governance processes (Edelenbos and van Meerkerk, 2015; Klijn et al., 2010; Stern and Coleman, 2015), we deem it important to reveal to what extent applied studies focus on both possible roles of trust in the water governance context. Finally, as an indication of the basis for such directional claims, we investigate to what extent they are supported by reference to earlier research and analysis of empirical data present in the article.

## 3 Research design and methods

### 3.1 Systematic review

Although synthesizing qualitative and quantitative empirical findings on a particular topic has traditionally been the main focus of systematic reviews (Liberati et al., 2009), systematic reviews are also increasingly used to provide a first systematic inventory of emerging research fields that would benefit from the development of new research frameworks and more holistic conceptualizations (Fischer et al., 2021; Torraco, 2005). Given our purpose to provide a first systematic overview of how the rapidly growing literature on trust in water governance engages with ‘trust’ as a conceptual lens, analytical device, and empirical phenomenon, it is this more ‘explorative’ type of literature review which suits our interests best. This review relies on reproducible methods for identifying, evaluating, and synthesizing characteristics of completed work in a field (Fischer et al., 2021; Snyder, 2019), through which we aim for making this review systematic and critical in its appraisal of existing conceptualizations and research approaches.

### 3.2 Article selection

**Fig. 1:**
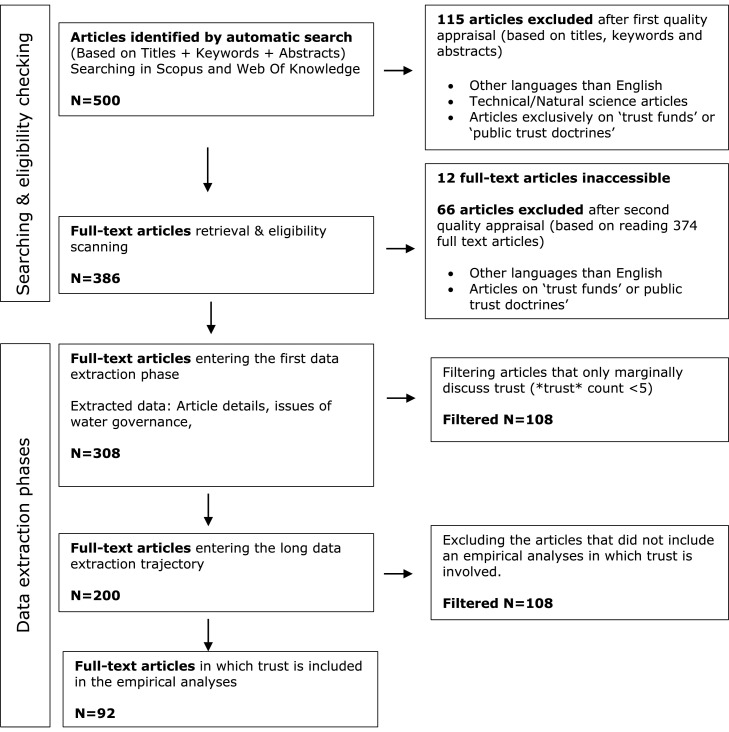
Flow Chart.

Our review started with an article selection procedure (the flowchart in Fig. 1 provides an overview of the entire article selection process). We first identified all articles of which the title, abstract or keywords suggest that both the concept of trust as well as the issue of water governance are captured. Using two scientific searching engines - Scopus and Web of Knowledge - we searched for articles in which the term *trust* (which also includes subsidiary terms such as ‘distrust’, ‘trustful’, and ‘trustworthy’) appears in combination with either one of the terms ‘water governance’, ‘water management’, or ‘water policy’.^1^ In January 2020, this search string obtained 500 articles that we subsequently subjected to a first screening round (based on the titles and abstracts) to identify and exclude off-topic articles. We excluded 115 articles that were mainly on the topics of ‘trust funds’, ‘public trust doctrines’, or articles with a technical focus from the natural sciences in which trust and water governance only incidentally appeared.

At the start of the second stage of our article selection process we obtained (with assistance of the libraries of our institutions) full-text access to 374 of the 386 articles that we retained after step one. We subjected those 374 articles to a second screening round (now based on the full-texts) after which we eliminated another 66 articles from our list that were off-topic or not written in English. Finally, we checked how often the term trust (or one of its derivatives) appeared in the 308 remaining articles. This check shows that in 30,5% of the 308 articles that we coded, the word trust (or one of its derivatives) appears less than five times. In other words, trust only plays a very marginal role in those articles. To focus our investigation about the role of trust in water governance to articles that deal substantially with the concept of trust, we limited our main analyses to the 200 articles in which the term trust appears at least five times.^2^

### 3.3 Data extraction

To analyze the 200 articles in our final sample, we developed a coding protocol with coding instructions (see online Appendix A). This protocol first covers questions to obtain basic article identification information. This includes questions on the type of journals publishing the articles, the dates of publication, whether the article is empirical or conceptual, and what sub-issue(s) of water governance is(are) addressed. To code the sub-issues within the field of water governance we first had a team discussion in which we identified ‘flood management’, ‘drought management’, ‘water quality management’, ‘water distribution management’, and ‘environmental conservation’ as the most likely sub-categories of water governance practices. We then coded to what extent the discussion in each article fitted into one or more of those categories or whether the issue should be classified as ‘other’.

We continued with a set of questions on the importance of trust in each article and its theoretical foundation. Subsequently, we identify the subjects and objects of trust that are discussed in each article. Although the subjects and objects of trust are commonly easier to identify in the cases in which trust is empirically studied, we also coded subjects of trust in cases in which they are only discussed in the more theoretical sections of articles. Furthermore, we allowed multiple entries as several subjects/objects of trust could simultaneously be discussed (and thus coded) in a single article. Some of the coded articles also use generic terms to refer to multiple subjects/objects of trust at the same time; such terms for example include inter-actor trust, stakeholder trust, and network trust. In cases that such generic terms appeared we always separately coded them as generic terms for several subjects/objects of trust. When articles went into further detail about the involved actors we additionally coded those more specific subjects of trust.

The next questions in the protocol ask about the geographic location and scale at which studies are performed and about the conclusions of the reviewed studies regarding the role of trust in water governance processes (*N* = 200). Finally, a last group of questions addresses how studies are performed and what methods have been used. Whereas the full sample of 200 articles contained many empirical articles (*n* = 164), we find that only a slight majority of 58% (*n* = 92) of the 164 empirical articles investigate the role of trust in water governance processes in their empirics. As our interest is only in the design and methods of studies that address trust in their empirics, we coded these methodological characteristics only for the sub-sample of 92 articles that empirically address trust.

Preliminary versions of the protocol have been tested and revised by several co-authors. All co-authors agreed on the final version of the codebook and subsequently coded their subset of articles. Thirty-seven articles were coded by two coders to determine intercoder agreement across non-text-based fields. Agreement of 80% or above was initially achieved across most of the variables with numerical answer categories (reported in appendix A). After discussions between the main coders, a few variables have been re-coded to reach this level of agreement. Questions that did not reach the 80% threshold level are not further discussed in our result section. The remaining text-based fields (e.g. the ‘definitions of trust’ and examples of ‘causal directions’) have been used to qualitatively inform our analyses. Data is made available in the supplements to this article.

## 4 Results

In this section, we first present a descriptive overview of the 200 articles in our sample and report which sub-issues of water governance are addressed by each article (4.1). Next, we show the spread of the sampled studies across geographies and scales (4.2), how trust is conceptualized (4.3), and what type of trust relations are most studied (4.4). Finally, we report how trust in water governance is empirically studied in the subset of 92 articles that contain such an analysis (4.5). To clearly distinguish between the articles in our sample (our primary data) and subsidiary literature used in this article, we refer to articles from our sample with their ID number in squared brackets. Online appendix B shows the bibliographical references belonging to these ID numbers.

### 4.1 Trust in the water governance literature: an emerging but dispersed field

Most of the 200 articles from our full dataset appeared in a broad selection of 106 different journals. Four articles appeared as conference proceedings while one article appeared as a book chapter. Individual journals which published five or more articles from our list are *Water* (13 articles), the *International Journal of Water Resources* (8 articles), *Environmental Science and Policy* (8 articles), *Ecology and Society* (6 articles), the *Journal of Environmental Management* (5 articles), the *Journal of Hydrology* (5 articles), and *Society and Natural Resources* (5 articles). A large majority of the 200 articles are empirical studies (82%). We classified the other articles as theoretical/review articles (13,5%), policy analyses (1,5%), case descriptions (1%), or ‘other’ (2%).

**Fig. 2:**
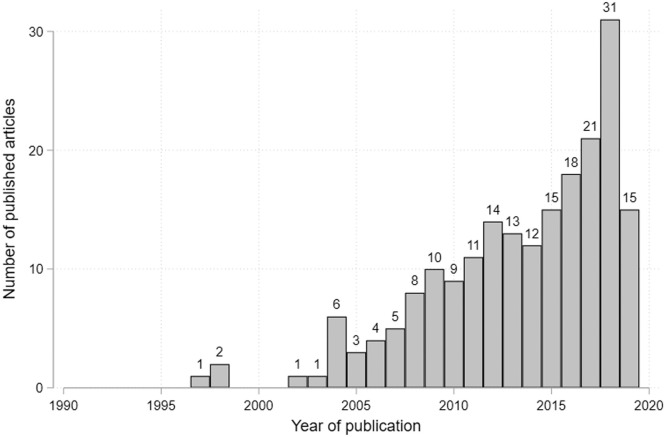
Published articles including trust and water governance by year (*N* = 200).

Fig. 2 shows that the number of annually published articles on trust in water governance is progressively increasing. Although the selected articles range over a time span from 1997 to 2019, only 20% of the 200 articles appeared before the year 2010 while 2018 has so far appeared as the most fruitful year with a total number of 31 published articles. Overall, those findings reassert our initial impression that the trust in water governance literature is in rapid development.

The results presented in table 1 reveal that there is substantial variation in how often different thematic sub-issues that fit under the generic label of water governance practices are addressed by the articles in our sample. A large majority of 70% of the 200 articles only deal with a single water governance sub-issue. Around 21% percent of the articles deal with two sub-issues while 10% of the articles simultaneously address three or more sub-issues of water governance. The sub-issues which are most addressed are ‘water distribution’ (addressed in 30% of all articles) and ‘water quality’ (29.5%). Other sub-issues such as ‘environmental conservation’ (15.5%), ’flood management’ (12%), and ‘drought management’ (10.5%) appear less frequently in the literature. Forty-seven percent of all the articles include a substantive issue that could only be classified into the ‘other water issues’ category. Interpretation of the text variable which describes those topics listed as ‘other’ shows that several of those articles deal with issues of transboundary water governance or with water governance in a general sense.

**Table 1:** Sub-issues of water governance.

Issues of water governance: (Multiple answers allowed)	(*N* = 200 articles) % (n)
Water distribution	30% (60)
Water quality	29.5% (59)
Environmental conservation	15.5% (31)
Flood management	12.0% (24)
Drought management	10.5% (21)
Other water issues	47.5% (95)

Number of issues addressed: (Single answer)	(*N* = 200 articles) (% (n)

- A single issue	70% (140)
- Two issues	21% (41)
- More than two	10% (19)

Total	100% (200)

### 4.2 Dominance of western geographies and studies at single scale

The examination of the spread across geographies and scales revealed two main patterns. First, the dataset shows a clear dominance of studies that cover Western geographies, notably Europe (22% of all the studies) and North America (21,5%). In addition, most of the studies that cover Oceania (12%) are in fact from Australia or New-Zealand. In contrast, there were relatively few studies from African (8%) or Latin American (6,5%) countries (Table 2). We also find that studies from these continents (and Asia) are cited less than half as many times as studies performed in Western geographies (table C2 in online appendix C). Recognizing the acuteness of water related issues in Africa and Latin America (Olagunju et al., 2019; Trimble et al., 2021), this indicates a considerable mismatch in scholarly attention. Having said that, we have to take into account that we focused on studies in English, as such we have not included studies in Spanish or French, both important languages in the global south. Second, a clear trend emerged in that studies tend to focus on a single geographical scale. For instance, 77% of the studies investigated water governance issues within a single country and 46% of the studies examined issues from a single region or watershed within a country. Only a limited number of the articles adopted cases based on a region or watershed that crosses international borders (8.5%), or cross-country comparative approaches based on comparing local (5.5%) or regional (7.5%) case studies from different countries (Tables 2 and 3).

**Table 2:** Geographic locations.

Geographic Location: (Single answer)	(*N* = 200 articles) % (n)
Europe	22% (44)
North America (Canada-US-Mexico)	21.5% (43)
Asia	18.5% (37)
Oceania (Australia-NZ-Solomon)	12% (24)
Africa	8% (16)
Central & South America	6.5% (13)
Multiple continents	8.5% (17)
None	3% (6)
Total	100% (200)

**Table 3:** Geographic scale.

Geographic scale of investigation: (Single answer)	(*N* = 200 articles) % (n)
A single region or watershed (single country)	45.5% (91)
Local, community, village, neighborhood (single country)	15.5% (31)
National level (single country)	12% (24)
Cross-border/international	8.5% (17)
Comparative: Regional issues from different countries	7.5% (15)
Comparative: Local issues from different countries	5.5% (11)
Other (specified in text)	1% (2)
Not applicable	4.5% (9)
Total	100% (200)

Additional investigation of how the sub-issues of water governance are spread over the different geographies that we distinguished reveal several interesting patterns in how the thematic focus of studies from different areas considerably varies (table C7 in online appendix C). Trust in relation to flood management is for example typically studied in the European context. Half of all the articles on trust in flood management are from European cases. Flooding is not, or hardly ever studied in relation to trust in studies that focus on Africa, Latin America, and Oceania. At the other hand, the issue of drought management and trust is hardly studied in the European context, which is surprising given the climatic trend of dryer and hotter summers in the continent which causes extensive problems for agriculture and water distribution (Grillakis, 2019). Studies on trust in relation to water quality issues most commonly appear in the North American context while the dominant focus in articles from Asia and Africa is on the issue of water distribution. Finally, an important insight is that although trust in water related environmental conservation is often studied in the Western context, this sub-issue is hardly ever studied in southern contexts (Africa, Asia, Latin America).

### 4.3 Limited conceptual clarity and an emphasis on the *instrumental* role of trust

A key finding from our review is that, overall, the available body of research on trust in water governance suffers from limited conceptual clarity. Only 11.5% (*n* = 23) of the articles included an explicit definition of trust and, of these, only 16 articles offered a reference to clarify the proposed conceptualization. Two sources are cited more than once, namely Hardin (2002) and Rousseau et al. (1998). Although only cited twice, the definitions in nine articles [IDs 62, 87, 109, 152, 152, 181, 225, 236, 271, 366] in essence come down to Hardin's basic understanding of a trust relationship (see Section 2) in which a subject of trust (A) trusts the object (B) concerning matters (X). Besides mentioning those three core components of a trust relationship, none of the definitions of trust in the mentioned articles include the elements of context specificity and the dynamic nature (timing) of trust (Bauer and Freitag, 2018; Lewicki et al., 2006). However, a few articles in fact do discuss the dynamic and context-specific nature of trust (see for example De Vries et al., 2017 [ID 87]; Marks and Zadoroznyj, 2005 [ID 234]), but did not incorporate such notions in their definitions of trust. Overall, our results show that the theoretical insights that trust relationships are often context-specific and change over time (Bauer and Freitag, 2018) are only very marginally incorporated in the literature on trust in water governance.

In addition, we find that half of the articles with explicit definitions of trust (*n* = 11) resonate with the view of Rousseau et al. (1998) that trust is a psychological state of a truster based upon *positive* expectations of the intentions or behavior of the trustee (albeit only in two cases with a cited reference to Rousseau) [IDs 109, 121, 152, 169, 181, 206, 225, 236, 250, 271, 332]. The other 12 articles that offer a definition of trust are neutral about what type of expectations trusters develop. The article by Cisneros (2019, p. 29 [ID 62]) for example simply states that trust is “the expectation that an individual has of the behavior of other stakeholders in a collaborative partnership”. Still, this suggests that, in those cases where trust is defined, the emphasis is often-times on its positive character.

A clear research question or goal related to trust appeared in only 17% (*n* = 33) of the 200 articles. Again, further interpretation identified a clear pattern in that about half of these articles stated a question or goal wherein the reason to engage with trust is primarily motivated due to *instrumental* reasons (i.e. enhancing trust is seen as a strategy to achieve other objectives (Olsen, 2006; Steen and Rutgers, 2011), which stands in contrast to, for instance, studies that focus on trust for its intrinsic value). For example, several articles focus on how to build trust in water governance practices [e.g. IDs 6, 45, 61, 152, 272] or how trust can increase the acceptance of certain water policies or technologies [e.g. IDs 11, 111, 120, 233, 234, 367]. The other half of the articles posed more descriptive questions, without any explicit view on the presumed role of trust.

Only 16% (*n* = 32) of the articles distinguish between different subtypes of trust. The subtype of trust that is most commonly mentioned is institutional trust, mostly to distinguish this type of trust from interpersonal trust [IDs 50, 156, 159, 180, 181, 308, 346, 378]. A few other articles apply a distinction between institutional trust and other more particular types of trust, such as trust in actual officeholders/administrations (sometimes labelled as political trust) [IDs 45, 104, 158, 169, 330, 380]. In addition, only a few articles in the review actively mention (but do commonly not operationalize and test) a distinction between antecedent based subtypes of trust; such as dispositional trust, calculative trust, and affinity based trust [IDs 104, 117, 181, 225, 236, 271, 276, 366]. In spite of the mentioned efforts to more extensively conceptualize trust, overall our findings show that most articles deal with trust as a single umbrella concept that refers to various social relations and actors.

Finally, when assessing the conceptual clarity of articles within each of the different sub-issues of water governance, we find that the term ‘trust’, on average, appears significantly less often in articles on flood prevention and nature conservation than in articles on the other issues. Furthermore, trust is hardly ever defined in the areas of drought management and water quality management, and distinctions between subtypes of trust hardly ever occur in articles on flood management and drought prevention (table C8 in online appendix C). When comparing between continents, we find that definitions of trust occur relative the least in papers on cases from North America, Asia, and Latin America. Subtypes of trust are the least distinguished in cases from Asia, Oceania, and Latin America while research questions on trust appear less often in papers dealing with Asian cases (table C9 in online appendix C). Nevertheless, we do not see a clear division between articles from Northern and Southern contexts when it comes to the conceptual clarity of the papers. With regard to the geographical scales of the investigations we find that trust is less often defined in cross-border and comparative papers than in case studies on the local, regional, or national scale. Cross-border studies and comparative studies that focus on regions also lag behind when it comes to distinguishing subtypes of trust and adopting research questions involving trust (table C10 in online appendix C).

### 4.4 Trust relations: a focus on trust of the general public in government organizations

While the articles in our sample exhibited considerable diversity regarding the trusters (subjects) and trustees (objects) under study (table 4), and associated trust relations (table 5), some patterns emerged. Trust that ordinary citizens hold is by far the most prevalent focus when it comes to the subjects of trust (appears in 49% of the articles). Individual farmers (26%), water managers (17%) and individual government employees (16%) are also in focus as subjects of trust. At the level of collectively held trust orientations, the entities that are most often discussed as trusters are (local and national-level) government organizations (25%). Other collectively held trust orientations are less often studied. It is noteworthy how social groups that tend to find themselves marginalized in water governance, such as ethnic minorities and indigenous peoples (e.g. Hoogesteger, 2012; Wester et al., 2003), are little represented as the subjects of trust in studies on the role of trust in water governance.

**Table 4:** Subjects & Objects of Trust.

Subject of Trust (Truster) (multiple answers allowed)	% of articles in which this subject is mentioned (*N* = 200 articles)	Object of Trust (Trustee) (multiple answers allowed)	% of articles in which this object is mentioned (*N* = 200 articles)
1) Individuals:		1) Individuals:	22% (44)
A) Ordinary citizens	49% (97)	2) Social groups: (minority/indigenous/religious groups)	16% (31)
B) Farmers	26% (52)	3) Private companies/firms:	16% (32)
C) Environmentalists	8% (16)	4) NGO's:	20% (40)
D Government employees/Civil servants	16% (32)	5) Governmental organizations:	
E) Water managers	17% (33)	A) Regional and local public bodies responsible for water management?	57% (113)
F) ‘Other’ individuals	7% (14)	B) National agencies responsible for water management?	34% (67)
2) Social groups:		C) National/Federal Governments	33% (65)
A) Farmer organizations	10% (20)	D) Supranational governments (EU, UN, NATO)	3% (6)
B) Environmental groups	9% (18)	6) Trust in formal institutions or rules: (i.e. legislation and norms)	
C) Religious groups	1% (1)	A) Operating permits, municipal laws….	5% (10)
D) Minorities	3% (5)	B) National level (e.g. Swedish Environmental Code)	5% (10)
E) Indigenous groups	6% (12)	C) Supranational /EU level (e.g. the EU Water Framework Directive)	3% (5)
F) Other	10% (20)	7A) Trust in water related knowledge:	20% (39)
3) Private companies/firms:	13% (26)	7B) Trust in scientists:	5% (9)
4) NGO's:	13% (26)	8) ‘Other’:	12% (24)
5) Governmental organizations:	26% (51)		
6) Nation States	11% (22)		
7) ‘Other’	24% (48)		

Number of times ‘other’ is used to indicate a term designating multiple subjects of trust	14% (28)	Number of times ‘other’ is used to indicate a term designating multiple objects of trust	15% (29)
Total number of articles with various subjects of trust	50% (99)	Total number of articles with various objects of trust	59% (117)

**Table 5:** Trust relations.

What type of relations are studied? (Multiple answers allowed)	% of articles in which this type of relationship is mentioned (*N* = 200 articles)
1) Trust of individual citizens in other individual-level actors	20% (39)
2) Trust of individual citizens in non-state affiliated groups	22% (43)
3) Mutual trust relations between different non-state affiliated groups	15% (29)
4) Trust of individual citizens in government organizations	55% (109)
5) Mutual trust relations between non-state affiliated groups and government organizations	28% (56)
6) Mutual trust relations between different government organizations	12% (24)
7) Trust relations between Nation States	6% (12)

Citizens (or individual-level actors) appear in 22% of the articles as the object of trust. This means that individuals are considerably less often studied as trustees than as trusters. As objects of trust, the articles that we coded primarily focus on trust in governmental organizations such as trust in local and regional governments (57%), national-level (executive) water management agencies (34%), and national-level government (policy-maker) (33%). Other group-level entities such as social groups (16%), private companies/firms (16%), and NGOs (20%) also commonly appear as the object of trust. Interestingly, only 3% of the articles paid attention to supranational government levels as objects of trust – something we find surprising, given the fact that many water-related policies today are developed at supranational levels (e.g. in the EU). Other objects of trust that rarely appear are trust in formal water management rules/laws/directives. Trust in water-related knowledge/facts is the last object of trust that is regularly mentioned (20%), while trust in scientists receives little attention (5%).

We furthermore assessed how often particular subject-object combinations appear to categorize the particular trust relations that are most commonly studied (table 5). We find that, by far, the most prevalent focus is on trust of individual citizens in government agencies (55%). Mutual trust relations between non-state affiliated actors at the group level (socially defined groups, private companies, and NGO's) and government organizations (28%), trust of individual citizens in non-state affiliated actors at the group level (22%), and trust of individuals in other individuals (20%) are also commonly addressed. Trust relations that are not so commonly studied are trust between different non-state affiliated group-level actors (15%), trust of government organizations in other government organizations (12%), and finally trust between nation states (6%).

We also note a considerable diversity in the literature when it comes to the number of specific trust relationships that are addressed in the articles. A first type of article takes a broad approach by focusing on multiple reciprocal trust relations between a set of different subjects and objects of trust. Several of those articles (15% of all articles) do not explicitly describe the particular subjects and objects of trust but rely upon more generic (and also more imprecise) terms such as *inter-agency trust, stakeholder trust*, or *network trust* to refer to the entire set of trust relations in multi-actor constellations. Among the articles that do not adopt such generic terms, we still find several articles that in fact address multiple (i.e. more than one) subjects (50%) or objects (59%) of trust. On the other hand, there is also a sizeable set of articles (41%) with a focus on a single unidirectional trust relation that only addresses the trust of a particular truster in a single type of trustee.

Additionally, we find substantial variation in the specific trust relations (and the various subjects and objects of trust) when separately investigating those relations within the thematic sub-issues of water governance. The most notable findings regarding the *subjects* of trust are that individual citizens are highly prevalent in the sub-issue of water quality management (64%) while they are comparatively understudied in the subfield of drought management (14%). Farmers as the subject of trust are relatively important in the fields of drought management (29%) and water distribution (35%), while water managers often appear in most sub-issues except for drought management (10%) and water quality management (7%). Indigenous populations and other non-indigenous minority groups do seldom play a role as subjects of trust. And when they do, they mainly play a role in the issue of nature conservation (in 13% of the articles on this issue).

Another finding is that individuals as the *object* of trust are less prominent than as the *subject* of trust: individual actors as objects of trust do not appear very often in the sub-issues of drought management (14%), water-quality management (14%), and nature conservation (10%). Furthermore, social groups as the object of trust are marginally studied in drought management. Civil society as the object of trust most commonly appears in the issue areas of flooding (29%) and nature conservation (29%). Supra-national governments as the objects of trust are only discussed in the issue areas of flooding, water-quality management, and nature conservation.

For the particular *trust relations* (specific subject-object combinations) we find that trust of individual trusters in individual trustees is relatively understudied within the sub-issues of water-quality management (9%) and nature conservation (13%). Relations between individuals and non-state affiliated groups get above average attention in the subfield of water-quality management (24%) while they are understudied in the subfield of drought management (10%). Trust of individual citizens in governmental actors (individual officeholders as well as institutions) is particularly well studied for the issues of water-quality management (58%) and nature conservation (54%). Relations between nation states are comparatively often studied in the fields of flooding (13%) and droughts (14%); while within the other subfields the percentages are below 7%.

### 4.5 Trust empirically studied: emphasis on trust as *explanatory* variable

Among the 92 articles that include an empirical assessment of the role of trust in water governance, the majority comprise of case study approaches (58%). Written surveys (55%) and oral interviews (51%) are the most adopted data collection methods. There was an almost even spread across quantitative (34%) and qualitative (27%) analyses, with a large part also combining qualitative and quantitative methods (38%). In terms of measuring the concept of trust, most of the studies posed questions that directly ask about a subject's level of trust (70%). Yet, a substantial number of 18% of the articles investigated trust by means of related concepts such as ‘satisfaction’ [ID 61] ‘the absence of conflicts’ [ID 15], ‘the willingness to co-operate’ [ID 1], or ‘legitimacy’ [ID 140]. For 12% of the articles that included an empirical assessment of trust, there was no account of how trust was actually measured. Overall, this shows that trust is, in about a third of the articles, not unequivocally operationalized, which should be considered when assessing whether the findings on trust are valid.

Moreover, we find that a large majority of the empirical findings on trust are centered on directional claims (92%), namely that trust explains, or is explained by, several other variables with which trust is associated (table 6). A few articles (8%) only report levels of trust as a result of an empirical investigation. In line with our earlier observation (in Section 4.3) about the oftentimes presumed instrumental role of trust, our review of the directionality of the empirically assessed trust claims points at an emphasis on trust as an explanatory variable (52%), i.e. as a variable that (positively) affects other water governance-related outcomes of primary concern such as participation and cooperation with projects and policies [IDs 81, 88, 128, 132, 180, 253, 291, 293, 330, 346, 351, 354, 376], behavioral adaptations (such as drinking desalinated water or water usage habits) [IDs 61, 133, 235, 238, 272, 289], adoption of environmental friendly water related techniques [IDs 3, 92, 158, 246, 261, 340, 344, 355], improved communication or social learning [IDs 62, 201, 269]). About one-fifth of the studies focus on trust as an outcome (18.5%). Identified variables that positively and/or negatively affect trust include the structural and social complexities of water governance issues [IDs 1, 8, 9, 157, 234, 236, 353, 339], levels of stakeholder involvement and collaborative efforts [IDs 1, 45, 56, 336], information procession and message framing [IDs 121, 130, 234, 236, 332, 339, 361, 381], and attitudes to risk [IDs 104, 116]. Fourteen articles (15%) investigate trust as both an outcome and an explanatory variable in their empirical analyses. Hurlimann [ID 162] for example simultaneously looks at the effect of the accurateness of information on trust in water recycling and the effect of trust on risk perceptions. Finally, another nine (10%) of the articles with directional claims deal with trust as a mediator/moderator/intermediate variable. Nancarrow, Leviston, Porter, and Tucker [ID 262] for example did not find a direct effect of trust on intended behaviors, but they found an indirect effect of trust due to its mediating role in the relation between risk assessments and behavioral intentions.

**Table 6:** The role played by trust in empirical analyses.

What type of (directional) claims do the empirical articles that involve trust make about the role played by trust?	(Total *N* = 92 % (n)
Trust Outcome	18.5% (17)
Trust Explanatory	52% (48)
Trust Outcome and Explanatory variable	15% (14)
Trust is mediator/moderator/intermediate variable	10% (9)
Non directional: Only level of trust assessed	4.5% (4)
Total	100% (92)

While we did not conduct any systematic quality assurance, we did investigate how the claims about trust were substantiated in the 92 studies. We find that quite a large number of 69 (75%) of the 92 articles demonstrate their main claim on the role of trust both with references to the existing literature as well as by means of their empirical analyses on trust. A smaller number of 16 (17%) of the 92 articles only rely on empirical findings to support their claims on trust. This level of substantiation in those 92 articles stands in strong contrast with the substantiation of the claims on trust in the 108 articles (from the entire set of 200 articles) that did not empirically investigate trust. In this latter group, claims on trust are only supported by means of references to existing literature, or not substantiated at all. This resonates with further comparisons of these groups; most notably that the level of conceptual clarity on trust is relatively better developed (although still often limited) in the 92 articles that contain empirical analyses involving trust.

## 5 Discussion

The research that elucidates the concept of trust and its importance in the context of water governance has expanded considerably since the early 1990s, with 80% of all articles on the subject having appeared since 2010. Nevertheless, our review revealed that the overall knowledge base has remained fragmented, which is in line with statements made about the state of the broader water governance literature as well (e.g. Pahl-Wostl, 2015, 2012).

Trust is a multi-dimensional concept that scholars have explored from very different angles, using different approaches. This makes it difficult to integrate different insights and to develop an all-encompassing theory of trust in water governance. Although diversity can also mean an enrichment of the literature, it currently mainly reflects the elusive nature of trust and hence the challenges of advancing the theoretical and empirical understanding of trust. The papers included in this literature review show that trust is a key issue in many water governance practices, yet understanding its exact role and functioning, and developing integrated knowledge on how to understand trust in water governance requires more research.

In the sections below, we more thoroughly reflect upon the main findings of our systematic literature review and connect these to recommendations for advancing future research on trust in the field of water governance. Finally, we discuss the limitations of our own study and end the article with a few concluding remarks.

### 5.1 Discussion of the main findings in relation to future research needs

*5.1.1 Don't neglect the extant ‘conceptualization problem’*

Our review generally corroborates the claim that trust is poorly conceptualized in water governance research. With respect to our set of conceptual criteria (on definitions, research questions/goals, and subtypes of trust), we find that a vast majority (89%) of studies in our sample use the term ‘trust’ without adopting any explicit statements that define trust. Moreover, among the small group of articles that do in fact define trust, there is considerable diversity in conceptualizing trust (as was expected by Davenport et al., 2007; Lijeblad et al., 2009; Pahl-Wostl, 2015; Stern and Coleman, 2015). Only a dozen studies clearly acknowledge the relational nature of trust, while context-specific and/or dynamic elements of trust are not mentioned at all in any of the definitional statements on trust. Notwithstanding, we observed a few occasions in which those elements are discussed in theoretical sections of papers (e.g. De Vries et al., 2017 [ID 87]; Marks and Zadoroznyj, 2005 [ID 234]). Altogether, these findings show that studies on trust in water governance are falling behind on some of the current developments in the broader literature on trust (Bauer and Freitag, 2018; Lewicki et al., 2006). Future progress first requires that more studies define and conceptualize trust. Second, to provide more complete assessments of trust relationships, we recommend studies to keep up with the broader literature on trust and the broader water governance literature by means of clearly acknowledging (and empirically uncovering) the context-specific and dynamic nature of trust relationships (see also Lubell, 2007 [ID 225]).

In addition, our review also shows that only a very selective number of articles incorporate the concept of trust into their stated research questions. Although for some articles this may result from trust only being a concept of subsidiary concern, for other papers in our sample (i.e. those papers in which trust in fact plays a major role) this suggests that more careful attention could be given to the concept of trust in the framing of research goals and questions. Notably, most studies tend to assess trust as an umbrella term rather than looking at its different dimensions (Stern and Coleman, 2015). Hence, the lack of more extensively developed trust frameworks limits the ability to understand these different dimensions of trust, how they relate to each other, and how they affect, or are affected by, other aspects of water governance (Pahl-Wostl, 2015; Reiersen, 2019). We advise future studies to rely upon more extensively developed trust frameworks so that the effects of trust can be empirically assessed and understood with regard to some of its component parts. Such approaches may follow the lead of some of the articles that we consider as good practice examples; such as Lubell's (2007 [ID 225]) study that assesses the independent effects of different types of (generalized) trust on trust in specific (water) policies, Onencan et al.’s (2018 [ID 271]) study that distinguishes between (dis)trust and trustworthiness in a game-based approach to model cooperation in shared river basin collective action problems, or Jorgensen et al.’s (2009 [ID 181]) investigation of the interplay between institutional trust and inter-personal trust in explaining water use behavior.

#### 5.1.2 Pick up on the understudied role of trust in several sub-issue/geography combinations

Water governance studies have mostly focused on the role of trust in issues such as ‘water distribution’ (especially papers on water distribution for agricultural use) and ‘water quality’ (predominantly articles on public opinion on drinking water provision). We found that considerably less attention has been paid to the role of trust in issues such as ‘environmental conservation’, ’flood management’, and ‘drought management’. In terms of geographical locations on which extant studies have focused, we most prominently find that little research has yet been conducted on the role of trust in water governance in the global south (Africa, Asia, Latin America)*.* Although one might argue that some of these latter issues simply appear less often (especially in the context of the global south), and that the role of trust is also less relevant in these issues/contexts, we would argue that this is not necessarily the case and that the role of trust in water governance practices is understudied in the global south. Specifically for specific sub-issue/geography combinations, there are several examples of highly relevant water related issues from within these contexts that need to be governed in settings that require trust. A few examples include the recent water crisis in the city of Cape Town (Maxmen, 2018), massive flooding events in Mozambique, Malawi and Zimbabwe (Charrua et al., 2021), and the life-threatening droughts in Eastern Africa (Gebremeskel Haile et al., 2019). In the context of the global south, our review shows that more attention could particularly be paid to the role of trust in issues of ‘flood prevention’ and ‘environmental conservation’, which are issues that despite their common occurrence and relevance in these contexts are hardly ever studied in combination with trust. In the northern (especially European) context on the other hand, studies on the role of trust in drought management are currently underexplored. Finally, the findings on the geographical scales of studies suggest a need for more studies with a multi-level (international) focus and studies that, for example, compare a set of local or regional case studies from different contexts and/or countries. Given the numerous water governance issues that extent borders, studies that go beyond a single (national) case are surprisingly scarce. As the role of trust and the causal mechanisms associated with trust might well be different in these understudied contexts, we might miss out on several important theoretical insights, which makes paying more attention to these contexts all the more important.

#### 5.1.3 Towards a larger diversity of the subjects & objects of trust

In line with the fragmented nature of the field of water governance itself – in which numerous actors are involved in several different issues (e.g. Lubell and Lippert, 2011 [ID 223]; Woodhouse and Muller, 2017) - we find a considerable diversity regarding the trusters (subjects) and trustees (objects) that are discussed by the entire set of studies. Overall, one can see two different streams in the literature. One focusing on public trust in government and water managers, and the other focusing on trust between various collaborating actors within water governance. Both have a distinct focus and their own approach, yet both write about trust, and therefore some confusion can arise. The more traditional actors within water governance processes receive most of the scholarly attention. Governments (at the local, regional, and national scale) and specific water management organizations are the most common objects of trust in the studies in our sample. It could be relevant to extent this focus to the international level and analyze how different forms of trust impact the possibilities for the formulation and adaption of international policies as well as how trust plays a role in their implementation. That we also identified trust in water-related knowledge as one of the central objects of trust speaks to the importance of such knowledge in relation to legitimizing actions and enhancing credibility of specific actors (e.g. Mase et al., 2015 [ID 236]; Medema et al., 2014 [ID 256]).

The general public (individual citizens) most often appears as the subject of trust. Much less attention is paid to how trust levels differ between groups within society, while the experiences and trust development of marginalized groups in societies, including ethnic minorities and indigenous peoples, hardly gain attention. In addition, given the scale of some of the water related challenges that water governance faces, supranational government levels as objects of trust also deserve more scholarly attention.

In terms of subject-object combinations, more attention is required to studies that look at trust relations between different non-state affiliated group-level actors, trust of government organizations in other government organizations, and finally trust between nation states. In addition, the relation between trust in governments and trust between actors involved in collaborative networks requires more attention, as participatory and collaborative processes are often initiated to enhance trust in government. Both concern different dimensions of trust, and drawing on the literature, little is known about how these relate to each other.

Finally, we identified a substantial subgroup of articles that rely upon generic terms to indicate trust relationships such as inter-agency trust, stakeholder trust, or network trust. However, several of these articles do not specify who the particular stakeholders and/or actors are who participate in such networks. To be able to more precisely understand how overall network performances are affected by the trust relations between its members, we recommend future studies to more clearly identify the involved subjects and objects of trust in networks and to more completely assess such trust relations (see for example Hickey et al., 2021; Song et al., 2017).

#### 5.1.4 Going beyond instrumentally motivated reasons to studying trust

Although it is theoretically expected that trust may manifest itself as a predictor as well as an outcome of water governance processes (Edelenbos and van Meerkerk, 2015; Klijn et al., 2010; Stern and Coleman, 2015), our findings show that the extant literature particularly focusses on approaching trust as an explanatory variable. This focus on trust as an explanatory variable comes together with a tendency in several of the articles that we analysed to assume that trust is an attitude which comes with positive consequences for establishing sustainable (long-term) cooperation in (water governance) processes that require collective action (Hamm et al., 2013; Lafuente et al., 2018 [ID 206]; Lubell, 2007 [ID 225]; Stern and Baird, 2015; van Meerkerk and Edelenbos, 2014). A textual analysis of the articles with stated research questions/goals and of the content of the directional claims that have been made on trust further revealed the omnipresence of instrumentally motivated reasons to engage with trust. For example, half of the articles with a clearly specified research question or goal related to trust already state in their introduction sections that they are mainly interested in seeking out how trust can increase acceptance of specific policies, governance practices, or technologies. Although not necessarily a problem in all cases, we agree with authors that argue that an overtly instrumental focus on trust can obscure the importance of trust building as an end in itself (Rutgers and Schreurs, 2006; Steen and Rutgers, 2011). When there is no up-front commitment to the process of trust building itself, collaborative processes may very well backfire into a loss of trust in case of any unwanted, negative outcomes of the practices that initially needed trust to be established (Ansell and Gash, 2007). Hence, we recommend paying more attention to trust as an intrinsically valuable outcome of water governance processes.

From an empirical perspective, we do not dispute that trust in several occasions may indeed play the presumed positive role (we found many examples of papers that report positive effects of trust on collective action and collaboration (e.g. Baldwin et al., 2018 [ID 17]; Hoogesteger, 2013 [ID 153]; Jorgensen et al., 2009 [ID 181])). Nevertheless, the results of our review warrant that we should question the validity and reliability of the knowledge base behind many of such findings and the relevance of such statements. Many of the claims on trust in water governance are not empirically assessed, and in cases in which they are, a poor conceptualization of trust in combination with methodological problems to assess trust undermines the validity of discussions on trust. Furthermore, among the articles that did empirically assess the role of trust in water governance, some of them in fact suggest that the positive effects of trust may be overrated as cooperation can, under certain conditions, occur without trust (Satein and Weber, 2018 [ID 308]) and higher trust does not always increase actors’ willingness to contribute to environmental common goods (e.g. Franzen et al., 2016 [ID 120]; Hanemann, 2014 [ID 139]) In addition, trust building is not always a relevant result of stakeholder involvement processes (e.g. Al Adwan and Hayek, 2011 [ID 5]; Buchecker et al., 2013 [ID 45]). Finally, our results also raise the question of whether the assumed beneficial effects of trust equally apply to all types of trusters and trustees. For example, as we have argued above, minorities and indigenous groups are scarcely represented as subjects of trust. This is a significant finding since individuals from these groups also tend to find themselves marginalized in water governance (e.g. Hoogesteger, 2012; Wester et al., 2003).

#### 5.1.5 Embrace methodological diversity

Our finding that the majority of the empirical assessments on the role of trust in water governance comprise of individual case study approaches is not surprising given that individual case studies abound in the larger water governance literature (Pahl-Wostl, 2015). To capture more of the complexities of water governance processes, we advocate that comparative approaches are more often adopted (e.g. Pahl-Wostl and Lebel, 2011). Such approaches may consist of (or combine) exploratory analyses that look at a large number of variables from multiple cases or(and) in-depth studies of selected cases that focus on a reduced number of variables only (Pahl-Wostl, 2015, p. 198). There is also a need for more studies with an international focus and for comparative studies that compare a set of local or regional case studies from different contexts and/or countries. Furthermore, although we endorse the substantial variation that exists when it comes to the methods of data collection/analyses, we observed that participatory methods are hardly applied in the field.

### 5.2 Limitations of our systematic review approach

There are some methodological limitations of our review approach. Given our searching procedure, we may have missed some unidentified gray literature on trust in water governance as well as non-English publications. Nevertheless, we are confident that the sample of articles that we analyzed is representative for the most substantial part of the trust in water governance literature as we coded the full collection of (English language) academic articles on the topic. Furthermore, some of the protocol development, coding, and interpretation of the findings was informed by the prior experiences and knowledge that our international group of authors brought to this project. Although such prior knowledge is inevitable in research, and an requirement to guide the methodological process of developing and performing the review, it also means that some of the categorizations and interpretations remain selective and non-exhaustive (Fischer et al., 2021). Finally, our choice of focusing on articles that mention the term *trust* (or one if its derivatives) at least five times indicates only a modest criteria for inclusion in the review. Although this choice fitted well with our aim of providing an overview of the way in which trust is discussed in the broader water governance literature, it could be argued that future work needs to focus more particularly on a smaller set of studies in which trust is the core concept of the contribution.

There are also some limitations in terms of potentially relevant content that we did not assess. For example, a need to broaden our knowledge base may be warranted when it comes to understanding how diverse governance contexts affect the role of trust in more particular water governance issues. Generalized trust in government institutions and more particular direct trust in stakeholders in water governance issues are only sparingly distinguished, from each other, and their interrelation barely studied. Furthermore, we could also have assessed more fully the uncritical extrapolation of findings on trust from singular studies that do not recognize the role of contextual variables, such as political history, governance situation, and power relations.

## 6 Concluding remarks

This systematic literature review has presented an overview of the way in which water governance literature engages with ‘trust’ as a conceptual lens, analytical device and empirical phenomenon. The review revealed that the current knowledge base on the role of trust in water governance is fragmented, lacks conceptual clarity, and is contextually dispersed. This state of the literature makes attempts to synthesize towards a sophisticated understanding of the role of trust in the field of water governance difficult, if not impossible (e.g. Srinivasan et al., 2012; Woodhouse and Muller, 2017). A key insight from our review is that future research would contribute towards a more comprehensive and useful understanding of trust in water governance by applying definitions and conceptualizations of trust that clearly acknowledge the context-specific and dynamic nature of trust relationships. By relying on clear and transparent conceptualizations, it is possible to empirically assess various aspects of trust, including factors that influence it, its possible effects, as well as the relationships between subjects and objects of trust. We thus foresee that future research could provide relevant and comparable knowledge on trust in water governance within the boundaries of well-specified (context) conditions - i.e. similarity between issues/geographies, comparable conceptualizations of trust, and a focus on similar subject/object combinations.

The analysis and information provided by our review should be of practical relevance for such a research effort since our database and appendices make it possible to identify studies with similarities in terms of the involved conditions, contexts, and subject/object combinations of particular trust relations, which enhances the possibilities of context specific comparisons and comparable empirical work. A final take home message for researchers and practitioners in the field is to critically assess the role and function of trust in water governance, and not assume that it will automatically play a positive role, since we found limited well-grounded empirical research supporting such claims.

## Declaration of competing interest

The authors declare that they have no known competing financial interests or personal relationships that could have appeared to influence the work reported in this paper.

## Data Availability

Data has been made available and is added to be published as an online file

## Appendix Supplementary materials

Image, application 1

Image, application 2

## Footnotes

1 We understand the concept of water governance in a broad sense so that it also refers to related (but sometimes more stringently defined) sub-concepts such as ‘water management’ and ‘water policy making’ (e.g. Pahl-Wostl, 2009). Technically, we used the following searching criteria: Topic = TITLE-ABS-KEY ("*trust*" AND "water governance" OR "water management" OR "water polic*"). No time limitations have been set for the period from which we retain articles.

2 We nevertheless coded the first thirteen questions from our protocol for the 108 articles in which trust appeared less than five times. The results show that trust indeed hardly plays a role in those articles. None of these articles comes up with a definition of trust neither does any of these articles adopt a conceptual distinction between different subtypes of trust.
