# Supplementary material for: A systematic review on the role of trust in the water governance literature
Source: Water Res X. 2022 Jun 27;16:100147. doi: 10.1016/j.wroa.2022.100147 (PMC9270239; doi:10.1016/j.wroa.2022.100147)
Supplement: Supplementary file 1 [file mmc1.docx]

**Appendix A: Codebook**

|  | **Coding fields** | **Decision rules** | **Type of entry** | **Percent agreement** |
| --- | --- | --- | --- | --- |
| **Basic Article Identification Variables** | | | | |
| 1 | Article ID (unique) |  | Text | - |
| 2 | Coder last name |  | Text | - |
| 3 | Author/s last name(s) |  | Text | - |
| 4 | Year of publication |  | Numeric | - |
| 5 | Journal |  | Text | - |
| 6 | Article title |  | Text | - |
| 7 | How often is the article cited? | Use the citation count statistic provided by Scopus/Web of Knowledge. | Numeric | - |
| **Basic Article Classification Variables** | | | | |
| 8a | What type of paper is this?*  1 = Conceptual/theoretical paper  2 = Empirical paper  3 = Review of literature  4 = Other type of paper (specify)  *It turned out that coders had difficulties to distinguish between 1 & 3. To increase the reliability, the lead authors collapsed the categories 1 & 3 into a single category. Furthermore, ‘policy analyses’ and ‘case description’ were often mentioned in the “other” category. We therefore listed them as specific answer categories.  The final recoded variable reads:  1 = Theoretical articles or reviews  2 = Empirical articles  3 = Policy analysis/recommendation  4 = Case description  5 = Other type of paper | -To qualify for code 2, the authors of an article should themselves have collected their data and/or present new results (when authors perform new analyses on existing data this also qualifies as empirical research).  -An article is a literature review (code 3) when it primarily discusses scientific articles. An article is more of a case overview (code 4 with ‘case description’ added as text) in case that it heavily relies on secondary articles which provide descriptive information on a case (such as geographical or historical information).  -When an article does not provide a systematic empirical analyses, but is more a collection of arguments based upon a few literature sources or a few interviews, code as ‘4’ other and specify for example as argumentative paper or journalistic paper. | Typology  +  Text if answer=4 | 81.1% (Based on the recoded variable) |
| 8b | Does the paper originate from a particular discipline? (law, history, political science, sociology, etc.) If yes, report the discipline. | -Only code this when it is obvious from the article that an article originates from a single specific discipline. | Text | Reliability below 80%. Not used in the article |
| 9 | Which sub-issues of water governance are primarily addressed? (More than 1 answer allowed)  1 = Flood prevention  2 = Drought prevention  3 = (Drinking) Water quality/pollution  4 = Water distribution  5 = Environmental/nature conservation  6 = Other (specify as comment) | -As multiple answers are allowed, all answer categories are provided as dummy (0/1) categories. Code every category that is applicable a ‘1’.  -In case you think that the topic of an article describes water governance in a very general sense, code as 6 and mention this in the comment section.  -If not made explicit in the text what aspect of water governance a paper is about, describe it in your own words. Do not use ‘not specified’. | Yes or no  +  Text if answer=6 | Average agreement over the five numerical answer categories = 84.6% |
| 10 | Is there a special reason or event that motivated this study?  1 = No: Ordinary period  2 = Natural disaster  3 = Economic crisis  4 = A specific policy process or political decision  5 = A specific process initiated by stakeholders (collaborative or resistance)  6 = Other (describe in text) | -Code 6 with as text reporting ‘climate change’ in case that authors clearly emphasize climate change as the motivation for their project.  -When articles talk about processes only in a very theoretical sense, this does not refer to a specific policy process. Code theoretical discussions of processes just as ‘ordinary period’ (Code 1). | Typology | Reliability below 80%. Not used in the article |
| **Trust related variables** | | | | |
| 11 | How often does the word *trust* (capturing distrust, trust, and trustworthiness) appear in the main text of the article. | -Use control F to search using *trust*. (Exclude the bibliography. Do not count if trust is in a header which is repeated at several pages of a publication).  -Do not manually add uncounted occurrences of *trust* from image file figures when these occurrences of trust have not been counted using the control F procedure. | Numerical | More than 80% of the articles deviate <=2 in a comparison of the reported trust count. |
| 12 | How Important is trust in the paper?  1 = Trust is presented as being theoretically or empirically one of the central concepts in this paper  2 = Trust is among a larger group of equally important concepts in this paper  3 = Trust plays only a minor role in this paper | -Code as 3 when *trust* occurs less than 5 times in a paper. Otherwise code as 2 or 1.  -Note: When trust occurs as ‘trust fund’ or ‘public trust doctrine’ this is not meaningful for us. We subtract those occurrences of trust from the total trust count. Example: when trust occurs 7 times but 3 of those occurrences are as ‘trust fund’, we consider this as 4 meaningful occurrences of trust. | Typology | 94.6% |
| 13a | Is *trust* defined? | -Explicit language must be used… Examples: ‘‘We define trust as…’’ ‘‘We see trust as…’’ ‘‘We borrow Smith’s definition of trust…’’ May be narrow or broad, and presented at any point in the article. The article must define trust, not just describe. | Yes or no | 94.6% |
| 13b | If question 13a = yes, provide a description of the definition. | -Quote text directly from article. | Text | - |
| 13c | In the case that trust is defined, which specific authors (or key publications) does this article refer to in its definition of trust? | -Mention at least the authors. In case a publication is mentioned in the main text of the article, also report this publication. | Text | - |
| 13d | Are subdivisions between different subtypes of trust made or mentioned at any place in the paper?  0 = No  1 = Yes | - Subtypes of trust that are distinguished in the literature are for example ‘dispositional trust’, ‘rational trust’, ‘affinitive trust’, ‘procedural trust’. Code this variable as ‘yes’ when such subtypes of trust are somewhere mentioned in the article that you code. | Yes or no | 83.8% |
| 13e | Make a decision on the further coding of the article:  1 = Long version (Proceed to 14a)  2 = Short version (Proceed to 24) | -If the answer to question 12=3 (the role of trust is only minor) and the answer to question 13a=0 (No), we adopt the short version of the coding procedure. In all other cases proceed with the long version of the coding procedure. | Typology | 100% |
| 14a | Is there a research question or goal related to ‘trust’ stated in the paper?  0 = No  1 = Yes | -Look at the introduction for direct evidence of an objective around trust. If uncertain mark no. | Yes or no | 91.9% |
| 14b | If yes, then what is the stated research question or goal related to trust? | -Quote text directly from the article or paraphrase. | Text | - |
| 15a | Who/what is the object of trust?  (More than 1 answer allowed)  1 = Individuals  2 = Particular social groups (neighborhood/minority/indigenous/  religious groups)  3 = Private companies/firms  4 = Civil society, e.g. NGO’s.  5 = Governmental organizations:  A) Regional and local public bodies responsible for water management? (E.g. Water District Authorities, County Administrative Boards and municipalities in Sweden)  B) National agencies responsible for water management? (E.g. Swedish Agency for Marine and Water Management)  C) National/Federal Governments  D) Supranational governments (EU, UN, NATO)  6 Trust in formal institutions or rules (i.e. legislation and norms regulating water use and management):  A) Operating permits, municipal laws….  B) National level (e.g. Swedish Environmental Code)  C) Supranational /EU level (e.g. Water Framework Directive, Habitats Directive and (dis)trust in energy/exploitation directed directives e.g. The Renewable Energy Directive?)  7 = Trust in water related knowledge/facts  8 = Other, specify | -In what/who do the subjects of this study trust?  -All answer categories are provided as dummy code categories. Code ‘1’ if a category is applicable. When a category is not applicable code ‘0’.  -In case the object of trust is a governmental organization at a specific level code as 5a or 5b or 5c etc. If the level of the governmental organization is unclear, just code as 5.  -In case the object of trust is a formal institution or rule, code as 6a or 6b or 6c if the level to which the institution applies is known. In case the level is unclear, just code as 6.  -(Step 1): In case that an article deals with trust in stakeholders/ network members/ actors in a general sense always use code 8 and write down in text the terminology used by the paper.  -(Step 2): In case that the paper also more specifically describes which type of actors/stakeholders it deals with, we additionally fill this out using the specific codes for each type actor mentioned in the text. When it is not mentioned which actors are part of a network, we only use code “8”, and provide zeros for all the dummy categories. | Yes or no  +  Text if other=yes | The average percent of agreement over the 12 answer categories is 83%. |
| 15b | Who are the subjects of trust?  (More than 1 answer allowed)  1 = Individuals:  A) Ordinary citizens  B) Farmers  C) Environmentalists  D) Government employees/  civil servants  E) Water managers  F) Other  2 = Social groups:  A) Farmer organizations  B) Environmental groups  C) Religious groups  D) Minorities  E) Indigenous groups  F) Other  3 = Private companies/firms  4 = NGO’s  5 = Governmental organizations  6 =Nation states  7 = Other, specify | - Who are the subjects who’s trust is studied?  -All answer categories are provided as dummy code categories. Code ‘1’ if a category is applicable. When a category is not applicable code ‘0’.  -In case that the subjects of trust are mentioned as a group of stakeholder/actor/network in general, follow the following procedure: (Step 1) Always apply code 7 “other” and write down in text the terminology used by the paper. (Step 2) In case that the paper also more specifically describes which type of actors/stakeholders it deals with, also apply the specific codes for each type of actor which is mentioned. When it is not mentioned which actors are part of a network, we only use code 7 and provide zeros for all the other categories. | Yes or no  +  Text if other=yes | The average percent of agreement over the 16 answer categories is 89%. |
| 15c | What type of relationships are studied? (More than 1 answer allowed)  1 = Between individual citizens  2 = Between individual citizens and non-state affiliated groups  3 = Between different non-state affiliated groups  4 = Between individual citizens and government organizations  5 = Between non-state affiliated groups and government organizations  6 = Between different government organizations  7 = Between Nation States | -Note: Social groups, Private companies, and NGO’s count as non-state affiliated groups.  -All answer categories are provided as dummy code categories in the excel sheet. Code ‘1’ if a category is applicable. When a category is not applicable code ‘0’. As more than one answer is allowed you may end up with more than one column that is coded as ‘1’. | Yes or no | The average percent of agreement over the 7 answer categories is 88%. |
| 16a | Does the author intend to empirically test the role of trust in the context of water governance?  0 = No  1 = Yes | -When the article includes an empirical analysis (which means code 2 is given in question 8a), but a trust is not dealt with in the empirics, code 16a as 0=No. When trust is incorporated in the empirical analyses code 16a as 1=Yes. | Yes or no | 81.1% |
| 16b | (Always code this question when 16a=1. When 16=0 this question should still be coded when an author makes claims about trust – in spite of not empirically testing those claims.)  What type of claim does the article make about the role played by trust?  1 = The paper primarily deals with trust as an outcome variable? (dependent variable)  2 = The paper primarily deals with trust as an explanatory variable (independent variable).  3 = Trust is both investigated as an outcome variable as well as an explanatory variable in this paper.  4 = Trust is primarily investigated as a mediator /moderator/intermediate variable in this study.  Note: (When 16b=3 both the items 16c & 16d should be coded if possible) | -Note: Trust as a *moderator* variable means that one’s level of trust affects the direction and/or strength of the relation between another independent variable X and dependent variable Y (such a relationship is also known as an ‘interaction effect’).  -Trust as a *mediator* variable means that trust in fact (partly) explains the observed relationship between two other variables X and Y. To observe mediation all the correlations between de independent variable X, the dependent variable Y, and the mediator ‘trust’ should be significant. When the effect of the mediator ‘trust’ is controlled for, the initially observed relation between X and Y should become weaker or disappear. | Typology | 86.7% (based on articles in which trust is included in the empirical analyses) |
| 16c | Which explicit factor(s) affect(s) trust?  (Code this item only if 16b=1 or 16b=3) | -Factors that explicitly influence *trust* according to the study results. Not theoretical or conceptual factors but the ones that authors actually discuss in their findings or conclusion sections.  -If an empirical study concludes that it remains unclear which determinants affect trust, this should also be reported. (May quote directly from the text). | Text | - |
| 16d | What is affected by trust and how?  (Code this item if 16b=2 or 16b=3) | -Only the effects the author actually discusses in the findings or conclusion section. Consider both what is affected and how, e.g. whether the change is positive or negative – using the language of the paper. (May quote directly from the text) | Text | - |
| **Questions on research Design**  (Code questions 17 & 20-25 only when 16a=1. Always code questions 18, 19 & 26) | | | | |
| 17 | Is trust directly measured or investigated by means of related concepts?  1 = Directly measured  2 = Related concepts | - If coded 2: name the related concepts. | Typology  +  Text if answer=2 | 80.0% (based on the set of 92 empirical articles) |
| 18 | Geographic scale of the empirical investigation:  0 = N/A;  1 = Local, community, village,  Neighborhood  2 = Region/watershed  3 = National;  4 = Cross-border/international (case is a single region/watershed which crosses borders)  5 = Other, please specify  6 = Multi-level: Several local cases from a single region/watershed  7 = Multi-level: Several regional case studies from a single country  8 = Multi-level: Within country (local or regional) cases from different countries. | -Categorize text according to typology. In case the answer = 1 or 2, add a description in which you name the village/region mentioned in the paper. In case that a number of lower level cases are clustered within a higher level case (e.g. villages within a region, regions within countries) code the lowest geographic scale as the basic scale of analyses. | Typology  +  text | 81.1% |
| 19 | Which country/countries are investigated? | -Name the countries. | Text | - |
| 20 | What is the main type of research design of the paper?  1 = Case study  2 = (Comparative) survey research  3 = Experiment/Game theory  4 = Theoretical argument  5 = Other (describe) | -Code the article as a case study (code 1) when the main focus of the paper is on investigating a certain sub-unit or sub-population from a larger collection of sub-units or a larger population in order to learn something about that more specific group or population.  -Code as code 2 ‘survey design’ when a survey has been used directed at an entire population of research units. This does mean that the research not deliberately focuses on a smaller sub-population of all research units (in that case it should be classified as a case study). In survey studies the focus is often on making generalizing statements about relationships at the individual level, not on the country as a case. | Typology  +  Text if answer=5 | 80% |
| 21 | Is the data collection quantitative or qualitative?  1 = Quantitative  2 = Qualitative  3 = Both quantitative and qualitative  4 = Other method(s) |  | Typology | 93.3% |
| 22 | What type of data collection is used? (More than one category allowed)  1 = Oral interviews,  2 = Written surveys,  3 = Document analyses,  4 = Focus groups/workshops  5 = Other (Name) |  | Typology  +  Text if answer=5 | The average percent of agreement over the 5 answer categories is 88%. |
|  |  |  |  |  |
| 23 | How many people or sub-units does the  author draw data from in this paper? | - What is the N of the study? Provide text to explain if the units of analyses are individuals, cases,  or other. NA = 0.  - A combination of units of analyses at different levels should both be coded. For example code as:[3 case studies at the regional level; approximately 50 survey respondents within each case]. | Quantity  +  Text | - |
| 24 | What is the overall take-away message  from the article in relation to trust? | -Briefly state the main conclusions or key insights. | Text | - |

**Appendix B**: **List of the 308 articles that** **entered the data extraction phase**

NOTE: There are 308 articles in this list but the associated article IDs run from 1 to 386 with 78 individual ID numbers not being listed. This is the case as the article IDs had already been provided to the initial set of 386 articles that we selected for a full-text retrieval. After the eligibility scanning round of the full text articles we excluded 78 more articles (that already received an ID) as ‘not accessible’ or ‘not on topic’ and only kept 308 articles to enter the first data extraction phase.

[ID=1] Abbink, K., Moller, L. C., & O’Hara, S. (2010). Sources of Mistrust: An experimental case study of a Central Asian water conflict. *Environmental and Resource Economics*, 45(2), 283–318. https://doi.org/10.1007/s10640- 009-9316-2

[ID=2] Adem Esmail, B., Geneletti, D., & Albert, C. (2017). Boundary work for implementing adaptive management: A water sector application. *Science of the Total Environment*, Volumes 593–594, 274–285. https://doi.org/10.1016/j.scitotenv.2017.03.121

[ID=3] Ahmed, A. E.-S. I., Hay, J. N., & El-Gohary, H. (2015). An investigation of the different factors affecting the adoption of water filters in South Mediterranean countries The case of Egypt. *Journal of Economic and Administrative Sciences*, 31(1), 2–19. https://doi.org/10.1108/JEAS-02-2014-0005

[ID=4] Akhmouch, A., & Correia, F. N. (2016). The 12 OECD principles on water governance – When science meets policy. *Utilities Policy*, 43, 14–20. https://doi.org/10.1016/j.jup.2016.06.004

[ID=5] Al Adwan, A., & Hayek, B. O. (2011). Participative irrigation management in the Jordan Valley. In WIT Transactions on Ecology and the Environment. Vol. 145, 537–546. https://doi.org/10.2495/WRM110471

[ID=6] Alexandra, J. (2019). Losing the authority–what institutional architecture for cooperative governance in the Murray Darling Basin? *Australian Journal of Water Resources*. https://doi.org/10.1080/13241583.2019.1586066

[ID=7] Al-Saèd, R., & Al-Hindi, A. M. (2012). Challenges of transboundary wastewater management for Palestinian communities along the Green Line - The Israeli- Palestinian border. In Shared Borders, Shared Waters: Israeli-Palestinian and Colorado River Basin Water Challenges (pp. 203–220). https://doi.org/10.1201/b13076

[ID=8] Al-Saidi, M., & Hefny, A. (2018). Institutional arrangements for beneficial regional cooperation on water, energy and food priority issues in the Eastern Nile Basin. *Journal of Hydrology*, 562, 821–831. https://doi.org/10.1016/j.jhydrol.2018.05.009

[ID=9] Alston, M., Whittenbury, K., Western, D., & Gosling, A. (2016). Water policy, trust and governance in the Murray-Darling Basin. *Australian Geographer*, 47(1), 49–64. https://doi.org/10.1080/00049182.2015.1091056

[ID=10] Alston, M., & Whittenbury, K. (2011). Climate change and water policy in Australia’s irrigation areas: A lost opportunity for a partnership model of governance. *Environmental Politics*, 20(6), 899–917. https://doi.org/10.1080/09644016.2011.617175

[ID=11] Ansink, E., Tesfaye, A., Bouma, J., & Brouwer, R. (2017). Cooperation in watershed management: A field experiment on location, trust, and enforcement. *Resource and Energy Economics*, 50, 91–104. https://doi.org/10.1016/j.reseneeco.2017.07.004

[ID=12] Apgar, J. M., Cohen, P. J., Ratner, B. D., de Silva, S., Buisson, M.-C., Longley, C., Mapedza, E. (2017). Identifying opportunities to improve governance of aquatic agricultural systems through participatory action research. *Ecology and Society*, 22(1). https://doi.org/10.5751/ES-08929-220109

[ID=13] Ashley, R. M., Newman, R., Walker, L., & Nowell, R. (2010). Changing a culture: Managing stormwater sustainably in the UK city of the future - Learning from the USA and Australia. (Conference paper). https://doi.org/10.1061/41099(367)135

[ID=14] Azhoni, A., Holman, I., & Jude, S. (2017). Contextual and interdependent causes of climate change adaptation barriers: Insights from water management institutions in Himachal Pradesh, India. *Science of the Total Environment*, Vol. 576, 817–828. https://doi.org/10.1016/j.scitotenv.2016.10.151

[ID=15] Babin, N., Mullendore, N. D., & Prokopy, L. S. (2016). Using social criteria to select watersheds for non-point source agricultural pollution abatement projects. *Land Use Policy*, Vol. 55, 327–333. https://doi.org/10.1016/j.landusepol.2015.06.021

[ID=17] Baldwin, E., McCord, P., Dell’Angelo, J., & Evans, T. (2018). Collective action in a polycentric water governance system. *Environmental Policy and Governance*, 28(4), 212–222. https://doi.org/10.1002/eet.1810

[ID=18] Ban, Y. U., Woo, H. M., Han, K. M., & Baek, J. I. (2013). Building an integrated governance model and finding management measures for nonpoint source pollution in watershed management of Korea. *Environmental Engineering Research*, 18(3), 199–208. https://doi.org/10.4491/eer.2013.18.3.199

[ID=19] Barbalios, N., Ioannidou, I., Tzionas, P., & Paraskeuopoulos, S. (2013). Behavioural adaptation towards efficient resource sharing under the lack of communication. *Journal of Environmental Informatics*, 21(2), 119–135. https://doi.org/10.3808/jei.201300239

[ID=20] Barber, M., & Jackson, S. (2012). Indigenous engagement in Australian mine water management: The alignment of corporate strategies with national water reform objectives. *Resources Policy*, 37(1), 48–58. https://doi.org/10.1016/j.resourpol.2011.12.006

[ID=22] Barua, A., & Vij, S. (2018). Treaties can be a non-starter: a multi-track and multilateral dialogue approach for Brahmaputra Basin. *Water Policy*, 20(5), 1027–1041. https://doi.org/10.2166/wp.2018.140

[ID=23] Bekchanov, M., Bhaduri, A., & Ringler, C. (2015). Potential gains from water rights trading in the Aral Sea Basin. *Agricultural Water Management*, 152, 41–56. https://doi.org/10.1016/j.agwat.2014.12.011

[ID=24] Berger, T., Birner, R., Díaz, J., McCarthy, N., & Wittmer, H. (2007). Capturing the complexity of water uses and water users within a multi-agent framework. Conference Paper. https://doi.org/10.1007/978-1-4020-5591-1-9

[ID=25] Bettini, Y., Brown, R., & De Haan, F. J. (2013). Water scarcity and institutional change: Lessons in adaptive governance from the drought experience of Perth, Western Australia. *Water Science and Technology*, 67(10), 2160–2168. https://doi.org/10.2166/wst.2013.127

[ID=26] Bhiremath, D. B., Shah, P., & Chaudhary, S. (2016). ICT interventions to improve the performance of canal irrigation sector in India. 03-06-June-2016. ACM International Conference Proceeding Series. https://doi.org/10.1145/2909609.2909635

[ID=27] Binder, L. C. W. (2006). Climate change and watershed planning in Washington State. *Journal of the American* *Water Resources Association*, 42(4), 915–926. https://doi.org/10.1111/j.1752-1688.2006.tb04504.x

[ID=28] Biswas, A. K. (2011). Cooperation or conflict in transboundary water management: Case study of South Asia. *Hydrological Sciences Journal*, 56(4), 662–670. https://doi.org/10.1080/02626667.2011.572886

[ID=29] Bizzi, S., Pianosi, F., & Soncini-Sessa, R. (2012). Valuing hydrological alteration in multi-objective water resources management. *Journal of Hydrology*, Vol. 472–473, 277–286. https://doi.org/10.1016/j.jhydrol.2012.09.033

[ID=30] Black, D. C., Wallbrink, P. J., & Jordan, P. W. (2014). Towards best practice implementation and application of models for analysis of water resources management scenarios. *Environmental Modelling and Software*, Vol. 52, 136–148. https://doi.org/10.1016/j.envsoft.2013.10.023

[ID=31] Blette, V. (2008). Drinking water public right-to-know requirements in the United States. *Journal of Water and Health*, 6(SUPPL. 1), 43–51. https://doi.org/10.2166/wh.2008.031

[ID=32] Bohman, A. (2012). The presence of the past: A retrospective view of the politics of urban water management in Accra, Ghana. *Water History*, 4(2), 137–154. https://doi.org/10.1007/s12685-011-0047-2

[ID=34] Bos, D. G., & Brown, H. L. (2015). Overcoming barriers to community participation in a catchment-scale experiment: Building trust and changing behavior. *Freshwater Science*, 34(3), 1169–1175. https://doi.org/10.1086/682421

[ID=35] Bosschaart, A., Kuiper, W., van der Schee, J., & Schoonenboom, J. (2013). The role of knowledge in students’ flood-risk perception. *Natural Hazards*, 69(3), 1661–1680. https://doi.org/10.1007/s11069-013-0774-z

[ID=36] Bots, P. W. G., Bijlsma, R., von Korff, Y., der Fluit, N., & Wolters, H. (2011). Supporting the Constructive Use of Existing Hydrological Models in Participatory Settings: a Set of ``Rules of the Game’’. Ecology and Society, 16(2).

[ID=37] Bourget, L., & Clamen, M. (1998). Managing extremes: The ijc experience. *Canadian Water Resources Journal*, 23(2), 135–142. https://doi.org/10.4296/cwrj2302135

[ID=42] Browning-Aiken, A., Richter, H., Goodrich, D., Strain, B., & Varady, R. (2004). Upper San Pedro Basin: Fostering collaborative binational watershed management. *International Journal of Water Resources Development*, 20(3), 353–367. https://doi.org/10.1080/0790062042000248574

[ID=45] Buchecker, M., Menzel, S., & Home, R. (2013). How much does participatory flood management contribute to stakeholders’ social capacity building? Empirical findings based on a triangulation of three evaluation approaches. *Natural Hazards and Earth System Sciences*, 13(6), 1427–1444. https://doi.org/10.5194/nhess-13-1427-2013

[ID=46] Burns, M. J., Wallis, E., & Matic, V. (2015). Building capacity in low-impact drainage management through research collaboration. *Freshwater Science*, 34(3), 1176–1185. https://doi.org/10.1086/682565

[ID=47] Cabello, V., Kovacic, Z., & Van Cauwenbergh, N. (2018). Unravelling narratives of water management: Reflections on epistemic uncertainty in the first cycle of implementation of the Water Framework Directive in southern Spain. *Environmental Science & Policy*, 85(SI), 19–27. https://doi.org/10.1016/j.envsci.2018.03.019

[ID=48] Cáñez-Cota, A., & Pineda-Pablos, N. (2019). Breaking out of the governance trap in rural Mexico. *Water Alternatives*, 12(1), 221–240.

[ID=50] Carlander, A., Von Borgstede, C., Jagers, S., & Sundblad, E.-L. (2016). A bridge over troubled water - public participation as a possibility for success in water management. *Water Policy*, 18(5), 1267–1285. https://doi.org/10.2166/wp.2016.225

[ID=51] Carr, G., Blöschl, G., & Loucks, D. P. (2014). Developing a dynamic framework to examine the interplay between environmental stress, stakeholder participation processes and hydrological systems. Conference proceeding. Retrieved from https://www.scopus.com/inward/record.uri?eid=2-s2.0-84904806236&partnerID=40&md5=1401b9f2346a8cd75c7d31584a6579b1

[ID=52] Carr, G., Blöschl, G., & Loucks, D. P. (2012). Evaluating participation in water resource management: A review. *Water Resources Research*, 48(11). https://doi.org/10.1029/2011WR011662

[ID=53] Chan, T., Ross, H., Hoverman, S., & Powell, B. (2010). Participatory development of a Bayesian network model for catchment-based water resource management. *Water Resources Research*, 46(7). https://doi.org/10.1029/2009WR008848

[ID=54] Chan, T., Powell, B., Hoverman, S., & Ross, H. (2008). Participatory approaches in developing a model to assist water resource management in a catchment in the Solomon Islands. 2, 850–857. Retrieved from https://www.scopus.com/inward/record.uri?eid=2-s2.0-84858314122&partnerID=40&md5=75eab08edae4d8361c9ebca1d5e1c4eb

[ID=55] Chanan, A., Kandasamy, J., Vigneswaran, S., & Sharma, D. (2009). A gradualist approach to address Australia’s urban water challenge. *Desalination*, 249(3), 1012–1016. https://doi.org/10.1016/j.desal.2009.09.012

[ID=56] Chandran, K. M., Joseph, E. J., & Sushanth, C. M. (2016). Performance evaluation of selected irrigation systems in Kerala State, India. Irrigation and drainage, 65(5), 613–619. https://doi.org/10.1002/ird.2012

[ID=58] Chen, S., Wang, Y., & Zhu, T. (2014). Exploring China’s farmer-level water-saving mechanisms: Analysis of an experiment conducted in taocheng district, hebei province. *Water (Switzerland),* 6(3), 547–563. https://doi.org/10.3390/w6030547

[ID=59] Chikozho, C. (2008). Stakeholder participatory processes and dialogue platforms in the Mazowe river catchment, Zimbabwe. *African Studies Quarterly*, 10(2–3), 27–44. Retrieved from https://www.scopus.com/inward/record.uri?eid=2-s2.0-77955840336&partnerID=40&md5=a257634a3db68f3cac609e27191795ea

[ID=60] Ching, L. (2016). A lived-experience investigation of narratives: recycled drinking water. *International Journal of Water Resources Development*, 32(4, SI), 637–649. https://doi.org/10.1080/07900627.2015.1126235

[ID=61] Christina Fragkou, M., & McEvoy, J. (2016). Trust matters: Why augmenting water supplies via desalination may not overcome perceptual water scarcity. *Desalination*, 397, 1–8. https://doi.org/10.1016/j.desal.2016.06.007

[ID=62] Cisneros, P. (2019). What makes collaborative water governance partnerships resilient to policy change? A comparative study of two cases in Ecuador. *Ecology and Society*, 24(1). https://doi.org/10.5751/ES-10667-240129

[ID=65] Clark, K. (2016). The Value of Water: The Flint Water Crisis as a Devaluation of Natural Resources, not a Matter of Racial Justice. *Environmental Justice*, 9(4, SI), 99–102. https://doi.org/10.1089/env.2016.0007

[ID=66] Clarvis, M. H., & Allan, A. (2014). Adaptive capacity in a chilean context: A questionable model for latin america. *Environmental Science and Policy*, 43, 78–90. https://doi.org/10.1016/j.envsci.2013.10.014

[ID=67] Clarvis, M. H., & Engle, N. L. (2015). Adaptive capacity of water governance arrangements: a comparative study of barriers and opportunities in Swiss and US states. *Regional Environmental Change*, 15(3), 517–527. https://doi.org/10.1007/s10113-013-0547-y

[ID=68] Closas, A., Molle, F., & Hernández-Mora, N. (2017). Sticks and carrots: Managing groundwater over-abstraction in La Mancha, Spain. IWMI Working Papers, Vol. 177. https://doi.org/10.5337/2017.218

[ID=69] Cockerill, K., Tidwell, V., & Passell, H. (2004). Assessing public perceptions of computer-based models. *Environmental Management*, 34(5), 609–619. https://doi.org/10.1007/s00267-003-0259-z

[ID=70] Conallin, J., Campbell, J., & Baumgartner, L. (2018). Using Strategic Adaptive Management to Facilitate Implementation of Environmental Flow Programs in Complex Social-Ecological Systems. *Environmental Management*, 62(5), 955–967. https://doi.org/10.1007/s00267-018-1091-9

[ID=71] Conallin, J. C., Dickens, C., Hearne, D., & Allan, C. (2017). Stakeholder Engagement in Environmental Water Management. In Water for the Environment: From Policy and Science to Implementation and Management (pp. 129–150). https://doi.org/10.1016/B978-0-12-803907-6.00007-3

[ID=72] Conrad, E. (2015). Bridging the hierarchical and collaborative divide: The role of network managers in scaling up a network approach to water governance in California. *Policy and Politics*, 43(3), 349–366. https://doi.org/10.1332/030557315X14350819637950

[ID=73] Cook, B. R., Atkinson, M., Chalmers, H., Comins, L., Cooksley, S., Deans, N., Spray, C. (2013). Interrogating participatory catchment organisations: Cases from Canada, New Zealand, Scotland and the Scottish-English Borderlands. *Geographical Journal*, 179(3), 234–247. https://doi.org/10.1111/j.1475-4959.2012.00492.x

[ID=75] Corrales, J., Naja, G. M., Bhat, M. G., & Miralles-Wilhelm, F. (2017). Water quality trading opportunities in two sub-watersheds in the northern Lake Okeechobee watershed. *Journal of Environmental Management*, 196, 544–559. https://doi.org/10.1016/j.jenvman.2017.03.061

[ID=76] Crampton, A., & Ragusa, A. T. (2016). Exploring perceptions and behaviors about drinking water in Australia and New Zealand: Is it risky to drink water, when and why? *Hydrology*, 3(1). https://doi.org/10.3390/hydrology3010008

[ID=80] Cundill, G., & Rodela, R. (2012). A review of assertions about the processes and outcomes of social learning in natural resource management. *Journal of Environmental Management*, 113, 7–14. https://doi.org/10.1016/j.jenvman.2012.08.021

[ID=81] Dare, M. (Lain), & Lukasiewicz, A. (2019). Are Environmental Water Advisory Groups an effective form of localism? *Journal of Environmental Planning and Management*, 62(2), 205–228. https://doi.org/10.1080/09640568.2017.1406343

[ID=84] De França Doria, M. (2010). Factors influencing public perception of drinking water quality. *Water Policy*, 12(1), 1–9. https://doi.org/10.2166/wp.2009.051

[ID=85] De Graaf, R. E., Dahm, R. J., Icke, J., Goetgeluk, R. W., Jansen, S. J. T., & Van De Ven, F. H. M. (2009). Receptivity to transformative change in the Dutch urban water management sector. *Water Science and Technology*, Vol. 60, pp. 311–320. https://doi.org/10.2166/wst.2009.179

[ID=87] De Vries, J. R., Van Bommel, S., Blackmore, C., & Asano, Y. (2017). Where there is no history: How to create trust and connection in learning for transformation in water governance. *Water (Switzerland)*, 9(2). https://doi.org/10.3390/w9020130

[ID=88] DeAngelo, M., & Nielsen-Pincus, M. (2017). Choosing the Right Policy Tools to Encourage Watershed Stewardship through the Study of Attitude. *Society and Natural Resources*, 30(11), 1328–1342. https://doi.org/10.1080/08941920.2017.1347973

[ID=89] DeCaro, D. A., Arnold, C. A. (Tony), Boamah, E. F., & Garmestani, A. S. (2017). Understanding and applying principles of social cognition and decision making in adaptive environmental governance. *Ecology and Society*, 22(1). https://doi.org/10.5751/ES-09154-220133

[ID=90] Di Matteo, M., Maier, H. R., & Dandy, G. C. (2019). Many-objective portfolio optimization approach for stormwater management project selection encouraging decision maker buy-in. *Environmental Modelling and Software*, 111, 340–355. https://doi.org/10.1016/j.envsoft.2018.09.008

[ID=91] Dillon, P., Bellchambers, R., Meyer, W., & Ellis, R. (2016). Community Perspective on Consultation on Urban Stormwater Management: Lessons from Brownhill Creek, South Australia. *WATER*, 8(5). https://doi.org/10.3390/w8050170

[ID=92] Dieu-Hang, T., Grafton, R. Q., Martínez-Espiñeira, R., & Garcia-Valiñas, M. (2017). Household adoption of energy and water-efficient appliances: An analysis of attitudes, labelling and complementary green behaviours in selected OECD countries. *Journal of Environmental Management*, 197, 140–150. https://doi.org/10.1016/j.jenvman.2017.03.070

[ID=93] DiMento, J. F. C. (2017). The Shape of Groundwater Law: California’s New Sustainability Act. *Journal of the Soutwest*, 59(1–2), 364–393. https://doi.org/10.1353/jsw.2017.0016

[ID=94] Distaso, A., & Ciervo, M. (2011). Water and common goods: Community management as a possible alternative to the public-private model. *Rivista Internazionale Di Scienze Sociali*, (2), 143–166. Retrieved from https://www.scopus.com/inward/record.uri?eid=2-s2.0-84862659864&partnerID=40&md5=63d433d7a1b8fd8ec5d63d59583f9ea9

[ID=95] Djumaboev, K., Hamidov, A., Anarbekov, O., Gafurov, Z., & Tussupova, K. (2017). Impact of institutional change on irrigation management: A case study from southern Uzbekistan. *Water (Switzerland)*, 9(6). https://doi.org/10.3390/w9060419

[ID=97] Dolfing, B. (2000). Sustainable water management in the Netherlands by water boards. *ICID Journal*, 49(4), 99–109.

[ID=98] Domenech, L., & Sauri, D. (2010). Socio-technical transitions in water scarcity contexts: Public acceptance of greywater reuse technologies in the Metropolitan Area of Barcelona. Resources Conservation and Recycling, 55(1), 53–62. https://doi.org/10.1016/j.resconrec.2010.07.001

[ID=100] Doyle, D. V. (1990). Water policy development in Manitoba: Meeting the challenge of the 90’s. *Canadian Water Resources Journal*, 15(2), 154–163. https://doi.org/10.4296/cwrj1502154

[ID=102] Drennan, M., Blum, C., & Whitman, E. J. (2000). The importance of a long range vision of the Los Angeles and San Gabriel Rivers Watershed Council. Conference Paper. https://doi.org/10.1061/40517(2000)128

[ID=103] Duda, A. M., & La Roche, D. (1997). Sustainable development of international waters and their basins: Implementing the GEF operational strategy. *International Journal of Water Resources Development*, 13(3), 383–401. Retrieved from https://www.scopus.com/inward/record.uri?eid=2-s2.0-0031225029&partnerID=40&md5=99885e817382573ec275d03708da50e2

[ID=104] Dudley, E., Walker, L., & Ashley, R. M. (2012). Socio-psychological behavioural aspects of learning and action alliances for water management and adapting to flood risk. Conference Paper. Retrieved from https://www.scopus.com/inward/record.uri?eid=2-s2.0-84862006003&partnerID=40&md5=aef9e9b52765725c45fa5c224e73fb79

[ID=105] Dunn, G., Brown, R. R., Bos, J. J., & Bakker, K. (2017). The role of science-policy interface in sustainable urban water transitions: Lessons from Rotterdam. *Environmental Science and Policy*, 73, 71–79. https://doi.org/10.1016/j.envsci.2017.04.013

[ID=106] Dupont, D., Waldner, C., Bharadwaj, L., Plummer, R., Carter, B., Cave, K., & Zagozewski, R. (2014). Drinking water management: Health risk perceptions and choices in first nations and non-First Nations communities in Canada. *International Journal of Environmental Research and Public Health*, 11(6), 5889–5903. https://doi.org/10.3390/ijerph110605889

[ID=107] Eberhard, R., Johnston, N., & Everingham, J.-A. (2013). A collaborative approach to address the cumulative impacts of mine-water discharge: Negotiating a cross-sectoral waterway partnership in the Bowen Basin, Australia. *Resources Policy*, 38(4), 678–687. https://doi.org/10.1016/j.resourpol.2013.02.002

[ID=109] Edelenbos, J., Van Meerkerk, I., & Van Leeuwen, C. (2015). Vitality of Complex Water Governance Systems: Condition and Evolution. *Journal of Environmental Policy & Planning*, 17(2), 237–261. https://doi.org/10.1080/1523908X.2014.936584

[ID=110] Ekstrom, J. A., Bedsworth, L., & Fencl, A. (2017). Gauging climate preparedness to inform adaptation needs: local level adaptation in drinking water quality in CA, USA. *Climatic Change*, 140(3–4), 467–481. https://doi.org/10.1007/s10584-016-1870-3

[ID=111] Emtage, N., & Herbohn, J. (2012). Implications of landholders management goals, use of information and trust of others for the adoption of recommended practices in the Wet Tropics region of Australia. *Landscape and Urban Planning*, 107(4), 351–360. https://doi.org/10.1016/j.landurbplan.2012.07.003

[ID=112] Enqvist, J. P., & Ziervogel, G. (2019). Water governance and justice in Cape Town: An overview. *Wiley Interdisciplinary Reviews-Water*, 6(4). https://doi.org/10.1002/wat2.1354

[ID=113] Evans, A. E. V, Giordano, M., & Clayton, T. (2012). Investing in agricultural water management to benefit smallholder farmers in Zambia: AgWater Solutions Project country synthesis report. IWMI Working Papers, Vol. 150. https://doi.org/10.5337/2012.212

[ID=114] Feldman, D. L., & Ingram, H. M. (2009). Making science useful to decision makers: Climate forecasts, water management, and knowledge networks. *Weather, Climate, and Society*, 1(1), 9–21. https://doi.org/10.1175/2009WCAS1007.1

[ID=115] Fenemor, A., Deans, N., Davie, T., Allen, W., Dymond, J., Kilvington, M., … Smith, R. (2008). Collaboration and modelling - Tools for integration in the Motueka catchment, New Zealand. *Water SA*, 34(4 SPEC. ISS.), 448–455. Retrieved from https://www.scopus.com/inward/record.uri?eid=2-s2.0-67649353149&partnerID=40&md5=a95f6f54967a06823e61882904f15e6a

[ID=116] Fielding, K. S., & Roiko, A. H. (2014). Providing information promotes greater public support for potable recycled water. *Water Research*, 61, 86–96. https://doi.org/10.1016/j.watres.2014.05.002

[ID=117] Fife-Schaw, C., Barnett, J., Chenoweth, J., Morrison, G. M., & Lundéhn, C. (2008). Consumer trust and confidence: Some recent ideas in the literature. Water Science and Technology: *Water Supply*, Vol. 8, pp. 43–48. https://doi.org/10.2166/ws.2008.035

[ID=118] Fischhendler, I., & Katz, D. (2013). The impact of uncertainties on cooperation over transboundary water: The case of Israeli-Palestinian negotiations. *Geoforum*, 50, 200–210. https://doi.org/10.1016/j.geoforum.2013.09.005

[ID=119] Fragkou, M. C., & McEvoy, J. (2016). Trust matters: Why augmenting water supplies via desalination may not overcome perceptual water scarcity. *Desalination*, 397, 1–8. https://doi.org/10.1016/j.desal.2016.06.007

[ID=120] Franzen, F., Dinnetz, P., & Hammer, M. (2016). Factors affecting farmers’ willingness to participate in eutrophication mitigation - A case study of preferences for wetland creation in Sweden. *Ecological Economics*, 130, 8–15. https://doi.org/10.1016/j.ecolecon.2016.05.019

[ID=121] Fraser, J., & Kunz, N. C. (2018). Water stewardship: Attributes of collaborative partnerships between mining companies and communities. *Water (Switzerland)*, 10(8). https://doi.org/10.3390/w10081081

[ID=122] Galvez, V., Rojas, R., Bennison, G., Prats, C., & Claro, E. (2019). Collaborate or perish: water resources management under contentious water use in a semiarid basin. *International Journal of River Basin Management*. https://doi.org/10.1080/15715124.2019.1634083

[ID=124] Ghosh, S., Sahoo, N., Verma, H. N., Singh, R., & Panda, D. K. (2004). Participatory water management for sustainable development in coastal belt of Orissa. *Journal of Rural Development*, 23(2), 217–229. Retrieved from https://www.scopus.com/inward/record.uri?eid=2-s2.0-3543079881&partnerID=40&md5=4912682e910d52f41623e4d6dda00843

[ID=126] Gill, B. C., Webb, J., Wilkinson, R., & Cherry, D. (2014). Irrigator responses to groundwater resource management in northern Victoria, southeastern Australia. *Journal of Hydrology*, 518(PA), 83–93. https://doi.org/10.1016/j.jhydrol.2014.04.046

[ID=128] Glenk, K., & Fischer, A. (2010). Insurance, prevB132:B136ention or just wait and see? Public preferences for water management strategies in the context of climate change. *Ecological Economics*, 69(11), 2279–2291. https://doi.org/10.1016/j.ecolecon.2010.06.022

[ID=129] Goldin, J. A. (2010). Water policy in South Africa: Trust and knowledge as obstacles to reform. *Review of Radical Political Economics*, 42(2), 195–212. https://doi.org/10.1177/0486613410368496

[ID=130] Goodwin, D., Raffin, M., Jeffrey, P., & Smith, H. M. (2018). Informing public attitudes to non-potable water reuse – The impact of message framing. *Water Research*, 145, 125–135. https://doi.org/10.1016/j.watres.2018.08.006

[ID=131] Gorton, M., Sauer, J., Peshevski, M., Bosev, D., Shekerinov, D., & Quarrie, S. (2009). Water Communities in the Republic of Macedonia: An Empirical Analysis of Membership Satisfaction and Payment Behavior. *World Development*, 37(12), 1951–1963. https://doi.org/10.1016/j.worlddev.2009.04.003

[ID=132] Gosnell, H., Chaffin, B. C., Ruhl, J. B., Arnold, C. A. (Tony), Craig, R. K., Benson, M. H., & Devenish, A. (2017). Transforming (perceived) rigidity in environmental law through adaptive governance: a case of Endangered Species Act implementation. *Ecology and Society*, 22(4). https://doi.org/10.5751/ES-09887-220442

[ID=133] Graymore, M., Wallis, A., & O’Toole, K. (2010). Understanding drivers and barriers: the key to water use behaviour change. *Water Science and Technology: Water Supply*, 10(5), 679–688. https://doi.org/10.2166/ws.2010.125

[ID=134] Grubert, E., & Cook, M. (2017). Communication science for science communication: Water management for oil and natural gas extraction. *Journal of Water Resources Planning and Management*, 143(11). https://doi.org/10.1061/(ASCE) WR.1943-5452.0000842

[ID=135] Hagemann, N., Klauer, B., Moynihan, R. M., Leidel, M., & Scheifhacken, N. (2014). The role of institutional and legal constraints on river water quality monitoring in Ukraine. *Environmental Earth Sciences*, 72(12, SI), 4745–4756. https://doi.org/10.1007/s12665-014-3307-5

[ID=136] Hall, D. M., Gilbertz, S. J., Anderson, M. B., & Ward, L. C. (2016). Beyond “buy-in”: designing citizen participation in water planning as research. *Journal of Cleaner Production*, 133, 725–734. https://doi.org/10.1016/j.jclepro.2016.05.170

[ID=137] Hanasz, P. (2017). Muddy waters: International actors and transboundary water cooperation in the Ganges-Brahmaputra problem shed. *Water Alternatives*, 10(2), 459–474. Retrieved from https://www.scopus.com/inward/record.uri?eid=2-s2.0-85020255606&partnerID=40&md5=b34d4e53bddba53b3fc9daf16cee6135

[ID=138] Hanasz, P. (2017). A Little less conversation? Track II dialogue and transboundary water governance. *Asia and the Pacific Policy Studies*, 4(2), 296–309. https://doi.org/10.1002/app5.183

[ID=139] Hanemann, M. (2014). Property rights and sustainable irrigation-A developed world perspective. *Agricultural Water Management*, 145, 5–22. https://doi.org/10.1016/j.agwat.2014.07.001

[ID=140] Harris, L. M. (2019). Assessing states: Water service delivery and evolving state–society relations in Accra, Ghana and Cape Town, South Africa. *Environment and Planning C: Politics and Space*. https://doi.org/10.1177/2399654419859365

[ID=141] Hartley, K. (2018). Environmental resilience and intergovernmental collaboration in the Pearl River Delta. *International Journal of Water Resources Development*, 34(4), 525–546. https://doi.org/10.1080/07900627.2017.1382334

[ID=142] Hartley, T. W. (2006). Public perception and participation in water reuse. *Desalination*, 187(1–3), 115–126. https://doi.org/10.1016/j.desal.2005.04.072

[ID=143] Hassenforder, E., Clavreul, D., Akhmouch, A., & Ferrand, N. (2019). What’s the middle ground? Institutionalized vs. emerging water-related stakeholder engagement processes. *International Journal of Water Resources Development*, 35(3), 525–542. https://doi.org/10.1080/07900627.2018.1452722

[ID=145] Heyns, P. (1995). The Namibian Perspective on Regional Collaboration in the Joint Development of International Water Resources. *International Journal of Water Resources Development*, 11(4), 467–492. https://doi.org/10.1080/07900629550042146

[ID=146] Hileman, J., Hicks, P., & Jones, R. (2016). An alternative framework for analyzing and managing conflicts in integrated water resources management (IWRM): linking theory and practice. *International Journal of Water Resources Development*, 32(5), 675–691. https://doi.org/10.1080/07900627.2015.1076719

[ID=147] Hill Clarvis, M., & Engle, N. L. (2013). Adaptive capacity of water governance arrangements: a comparative study of barriers and opportunities in Swiss and US states. *Regional Environmental Change*, 15(3), 517–527. https://doi.org/10.1007/s10113-013-0547-y

[ID=148] Hill, M. (2013). Adaptive capacity of water governance: Cases from the Alps and the Andes. *Mountain Research and Development*, 33(3), 248–259. https://doi.org/10.1659/MRD-JOURNAL-D-12-00106.1

[ID=149] Hofmann, N., & Mitchell, B. (1998). The RESPECT model: Evolving decision-making approaches in water management. *Water Policy*, 1(3), 341–355. https://doi.org/10.1016/S1366-7017(98)00028-2

[ID=150] Holley, C., Sinclair, D., Lopez-Gunn, E., & Schlager, E. (2016). Conjunctive management through collective action. In Integrated Groundwater Management: Concepts, Approaches and Challenges (pp. 229–252). https://doi.org/10.1007/978-3-319-23576-9_9

[ID=151] Hongze, T., Jing, L., Inge, O. G., Yan, Z., & Jiahua, P. (2015). The Pollution Caused by Protection: The Unintended Consequences of the Local Governance of the Urban Drinking Water Source Protection in Tianjin, China. *Chinese Journal of Urban and Environmental Studies*, 3(3). https://doi.org/10.1142/S2345748115500256

[ID=152] Hoogesteger, J. (2013). Trans-Forming Social Capital Around Water: Water User Organizations, Water Rights, and Nongovernmental Organizations in Cangahua, the Ecuadorian Andes. *Society and Natural Resources*, 26(1), 60–74. https://doi.org/10.1080/08941920.2012.689933

[ID=153] Hooper, B. P. (2008). Covenant action to facilitate integrated river basin management. *Water SA*, 34(4 SPEC. ISS.), 456–460. Retrieved from https://www.scopus.com/inward/record.uri?eid=2-s2.0-67649383764&partnerID=40&md5=28e5a5018c6b614ee4aea9b4d4da1bd4

[ID=155] Hove, J., D’Ambruoso, L., Mabetha, D., Van Der Merwe, M., Byass, P., Kahn, K., … Twine, R. (2019). “Water is life”: Developing community participation for clean water in rural South Africa. *BMJ Global Health*, 4(3). https://doi.org/10.1136/bmjgh-2018-001377

[ID=156] Hoverman, S., Ross, H., Chan, T., & Powell, B. (2011). Social learning through participatory integrated catchment risk assessment in the Solomon Islands. *Ecology and Society*, 16(2). https://doi.org/10.5751/ES-04036-160217

[ID=157] Hu, X.-J., Xiong, Y.-C., Li, Y.-J., Wang, J.-X., Li, F.-M., Wang, H.-Y., & Li, L.-L. (2014). Integrated water resources management and water users’ associations in the arid region of northwest China: A case study of farmers’ perceptions. *Journal of Environmental Management*, 145, 162–169. https://doi.org/10.1016/j.jenvman.2014.06.018

[ID=158] Hunecke, C., Engler, A., Jara-Rojas, R., & Poortvliet, P. M. (2017). Understanding the role of social capital in adoption decisions: An application to irrigation technology. *Agricultural Systems*, 153, 221–231. https://doi.org/10.1016/j.agsy.2017.02.002

[ID=159] Hurlbert, M., & Gupta, J. (2015). The split ladder of participation: A diagnostic, strategic, and evaluation tool to assess when participation is necessary. *Environmental Science and Policy*, 50, 100–113. https://doi.org/10.1016/j.envsci.2015.01.011

[ID=160] Hurlimann, A., Hemphill, E., McKay, J., & Geursen, G. (2008). Establishing components of community satisfaction with recycled water use through a structural equation model. *Journal of Environmental Management*, 88(4), 1221–1232. https://doi.org/10.1016/j.jenvman.2007.06.002

[ID=161] Hurlimann, A. (2007). Attitudes to future use of recycled water in a Bendigo office building. *Water*, 34(6), 58+60+62-64. Retrieved from https://www.scopus.com/inward/record.uri?eid=2-s2.0-39149143366&partnerID=40&md5=b0618cd5bb682ad6d16c502cca3c00c3

[ID=162] Hurlimann, A. C. (2007). Is recycled water use risky? An Urban Australian community’s perspective. *Environmentalist*, 27(1), 83–94. https://doi.org/10.1007/s10669-007-9019-6

[ID=164] Iyer, R. R. (2010). Governance of water: The legal questions. *South Asian Survey*, 17(1), 147–157. https://doi.org/10.1177/097152311001700111

[ID=165] Jackson, M., Stewart, R. A., Fielding, K. S., Cochrane, J., & Beal, C. D. (2019). Collaborating for sustainable water and energy management: Assessment and categorization of indigenous involvement in remote Australian communities. *Sustainability (Switzerland)*, 11(2). https://doi.org/10.3390/su11020427

[ID=166] Jackson, S., Tan, P.-L., & Nolan, S. (2012). Tools to enhance public participation and confidence in the development of the Howard East aquifer water plan, Northern Territory. *Journal of Hydrology*, 474(SI), 22–28. https://doi.org/10.1016/j.jhydrol.2012.02.007

[ID=167] Jackson, S. (2019). Building trust and establishing legitimacy across scientific, water management and Indigenous cultures. *Australian Journal of Water Resources*, 23(1), 14–23. https://doi.org/10.1080/13241583.2018.1505994

[ID=168] Jacobi, P. R., & Monteiro, F. (2006). Social capital and institutional performance methodological and theoretical discussion on the water casin committees in metropolitan São Paulo - Brazil. *Ambiente e Sociedade*, 9(2), 25–45. Retrieved from https://www.scopus.com/inward/record.uri?eid=2-s2.0-34249323411&partnerID=40&md5=223fec80e37b3306185c9e11b7f1895d

[ID=169] Jacobs, I. M. (2012). A community in the Orange: The development of a multi-level water governance framework in the Orange-Senqu River basin in Southern Africa. *International Environmental Agreements: Politics, Law and Economics*, 12(2), 187–210. https://doi.org/10.1007/s10784-012-9166-1

[ID=170] Jain, M., Lim, Y., Arce-Nazario, J. A., & Uriarte, M. (2014). Perceptional and Socio-Demographic Factors Associated with Household Drinking Water Management Strategies in Rural Puerto Rico. *PLOS ONE*, 9(2). https://doi.org/10.1371/journal.pone.0088059

[ID=171] Jalba, D. I., Cromar, N. J., Pollard, S. J. T., Charrois, J. W., Bradshaw, R., & Hrudey, S. E. (2010). Safe drinking water: Critical components of effective inter-agency relationships. *Environment International*, 36(1), 51–59. https://doi.org/10.1016/j.envint.2009.09.007

[ID=172] Jamati, C. (2003). Casablanca (Morocco): An example of public-private partnership. *International Journal of Water Resources Development*, 19(2), 153–158. https://doi.org/10.1080/0790062032000089284

[ID=174] Jenkins, B. R. (2018). Socio-economic issues and collaborative governance. *Global Issues in Water Policy*, Vol. 19, pp. 463–504. https://doi.org/10.1007/978-94-024-1213-0_14

[ID=175] Jensen, O., & Chindarkar, N. (2019). Sustaining Reforms in Water Service Delivery: the Role of Service Quality, Salience, Trust and Financial Viability. *Water Resources Management*, 33(3), 975–992. https://doi.org/10.1007/s11269-018-2154-7

[ID=176] Jetoo, S. (2019). An assessment of the baltic sea action plan (BSAP) using the OECD principles on water governance. *Sustainability (Switzerland),* 11(12). https://doi.org/10.3390/su10023405

[ID=180] Jones, N., Evangelinos, K., Gaganis, P., & Polyzou, E. (2011). Citizens’ Perceptions on Water Conservation Policies and the Role of Social Capital. *Water Resources Management*, 25(2), 509–522. https://doi.org/10.1007/s11269-010-9711-z

[ID=181] Jorgensen, B., Graymore, M., & O’Toole, K. (2009). Household water use behavior: An integrated model. *Journal of Environmental Management*, 91(1), 227–236. https://doi.org/10.1016/j.jenvman.2009.08.009

[ID=182] Kalcic, M. M., Frankenberger, J., Chaubey, I., Prokopy, L., & Bowling, L. (2015). Adaptive Targeting: Engaging Farmers to Improve Targeting and Adoption of Agricultural Conservation Practices. *Journal of the American Water Resources Association*, 51(4), 973–991. https://doi.org/10.1111/1752-1688.12336

[ID=183] Kamran, M. A., Aijaz, A., & Shivakoti, G. P. (2016). Institutions for Governance of Transboundary Water Commons: The Case of the Indus Basin. In Redefining Diversity and Dynamics of Natural Resources Management in Asia (Vol. 2, pp. 207–223). https://doi.org/10.1016/B978-0-12-805453-6.00013-9

[ID=185] Kastelan-Macan, M., Ahel, M., Horvat, A. J. M., Jabucar, D., & Jovancic, P. (2007). Water resources and waste water management in Bosnia and Herzegovina, Croatia and the State Union of Serbia and Montenegro. *Water Policy*, 9(3), 319–343. https://doi.org/10.2166/wp.2007.003

[ID=186] Katz, D., & Fischhendler, I. (2011). Spatial and temporal dynamics of linkage strategies in Arab-Israeli water negotiations. *Political Geography*, 30(1), 13–24. https://doi.org/10.1016/j.polgeo.2010.12.002

[ID=190] Kessler, C. A. (2008). Laying a solid foundation for sustainable development in Bolivian mountain villages. *Environment, Development and Sustainability*, 10(2), 233–247. https://doi.org/10.1007/s10668-006-9062-3

[ID=191] Keuls, C. (2008). Knowledge network development in water resources and irrigation management: The case of Cknet-Ina in Indonesia. *Irrigation and Drainage*, 57(3), 341–353. https://doi.org/10.1002/ird.428

[ID=192] Khan, S., & Mushtaq, S. (2009). Regional partnerships to assist public-private investments in irrigation systems. *Agricultural Water Management*, 96(5), 839–846. https://doi.org/10.1016/j.agwat.2008.11.007

[ID=193] Khan, S. J., & Gerrard, L. E. (2006). Stakeholder communications for successful water reuse operations. *Desalination*, 187(1–3), 191–202. https://doi.org/10.1016/j.desal.2005.04.079

[ID=194] King, A. B., & Thornton, M. (2016). Staying the course: Collaborative modeling to support adaptive and resilientwater resource governance in the inland northwest. *Water (Switzerland)*, 8(6). https://doi.org/10.3390/w8060232

[ID=196] Klaasse, A., Jarmain, C., Roux, A., Becu, O., & Ginati, A. (2011). Grapelook: Space based services to improve water use efficiency of vineyards in South Africa. 62nd International Astronautical Congress, 4209–4214. Retrieved from https://www.scopus.com/inward/record.uri?eid=2-s2.0-84864082593&partnerID=40&md5=34b9789c5d1965338d3c2b6262b347d9

[ID=197] Knamiller, C., & Sharp, L. (2009). Issues of trust, fairness and efficacy: A qualitative study of information provision for newly metered households in England. *Water Science and Technology: Water Supply*, 9(3), 311–319. https://doi.org/10.2166/ws.2009.433

[ID=198] Kolkman, M. J., Kok, M., & van der Veen, A. (2005). Mental model mapping as a new tool to analyse the use of information in decision-making in integrated water management. *Physics and Chemistry of the Earth*, 30(4-5 SPEC. ISS.), 317–332. https://doi.org/10.1016/j.pce.2005.01.002

[ID=199] Korjonen-Kuusipuro, K. (2011). Critical water: Negotiating the Vuoksi River in 1940. *Water History*, 3(3), 169–186. https://doi.org/10.1007/s12685-011-0035-6

[ID=200] Krywkow, J., & Hare, M. (2008). Participatory process management. Conference Proceeding, 888–899. Retrieved from https://www.scopus.com/inward/record.uri?eid=2-s2.0-84858306561&partnerID=40&md5=4d05e17229f324a275b610a7d3118944

[ID=201] Kumler, L. M., & Lemos, M. C. (2008). Managing waters of the Paraiba do Sul River Basin, Brazil: A case study in institutional change and social learning. *Ecology and Society*, 13(2). Retrieved from https://www.scopus.com/inward/record.uri?eid=2-s2.0-58749097827&partnerID=40&md5=1e1ccf92d30ed0736581c5cdddcd5dad

[ID=202] Kuzdas, C., & Wiek, A. (2014). Governance scenarios for addressing water conflicts and climate change impacts. *Environmental Science and Policy*, 42, 181–196. https://doi.org/10.1016/j.envsci.2014.06.007

[ID=203] Kuzdas, C., Warner, B., Wiek, A., Yglesias, M., Vignola, R., & Ramírez-Cover, A. (2016). Identifying the potential of governance regimes to aggravate or mitigate local water conflicts in regions threatened by climate change. *Local Environment*, 21(11), 1387–1408. https://doi.org/10.1080/13549839.2015.1129604

[ID=204] Lackstrom, K., Kettle, N. P., Haywood, B., & Dow, K. (2014). Climate-sensitive decisions and time frames: A cross-sectoral analysis of information pathways in the Carolinas. *Weather, Climate, and Society*, 6(2), 238–252. https://doi.org/10.1175/WCAS-D-13-00030.1

[ID=205] Lacroix, K. E. M., & Megdal, S. B. (2016). Explore, Synthesize, and Repeat: Unraveling Complex Water Management Issues through the Stakeholder Engagement Wheel. *WATER*, 8(4). https://doi.org/10.3390/w8040118

[ID=206] Lafuente, R., Paneque, P., & Vargas, J. (2018). The role played by environmental concern and institutional trust in changing public preferences for water management. *Environmental Policy and Governance*, 28(6), 441–452. https://doi.org/10.1002/eet.1808

[ID=208] Lalika, M. C. S., Meire, P., & Ngaga, Y. M. (2015). Exploring watershed conservation and water governance along Pangani River Basin, Tanzania. *Land Use Policy*, 48, 351–361. https://doi.org/10.1016/j.landusepol.2015.06.010

[ID=210] Langsdale, S. M., Beall, A., Carmichael, J., Cohen, S. J., Forster, C. B., & Neale, T. (2009). Exploring the implications of climate change on water resources through participatory modeling: Case study of the Okanagan Basin, British Columbia. *Journal of Water Resources Planning and Management*, 135(5), 373–381. https://doi.org/10.1061/(ASCE)0733-9496(2009)135:5(373)

[ID=211] Lanier, A. L., & Sukop, M. C. (2016). Interdisciplinary Projects Require an Adaptive and Agile Management Approach: South Florida Water, Sustainability, and Climate Project Experience (P. C.S. & R. D., Eds.). https://doi.org/10.1061/9780784479865.019

[ID=213] Leach, W. D. (2002). Surveying diverse stakeholder groups. *Society and Natural Resources*, 15(7), 641–649. https://doi.org/10.1080/08941920290069245

[ID=214] Leta, G., Kelboro, G., Stellmacher, T., Van Assche, K., & Hornidge, A.-K. (2018). Nikinake: the mobilization of labour and skill development in rural Ethiopia. *Natural Resources Forum*, 42(2), 93–107. https://doi.org/10.1111/1477-8947.12145

[ID=215] Letcher, R. A., Jakeman, A. J., & Croke, B. F. W. (2004). Model development for integrated assessment of water allocation options. *Water Resources Research*, 40(5), W055021–W0550215. https://doi.org/10.1029/2003WR002933

[ID=216] Lindsay, A. (2018). Social learning as an adaptive measure to prepare for climate change impacts on water provision in Peru. *Journal of Environmental Studies and Sciences*, 8(4), 477–487. https://doi.org/10.1007/s13412-017-0464-3

[ID=218] Lipchin, C., Best, J., Cutler, J., & Kronich, S. (2016). Mitigating Transboundary Wastewater Conflicts: Building Partnerships and Trust Through Collaborative Dialogue. *Journal of Peacebuilding and Development*, 11(2), 72–77. https://doi.org/10.1080/15423166.2016.1181001

[ID=219] Loomis, J. B. (2000). Environmental valuation techniques in water resource decision making. *Journal of Water Resources Planning and Management*, 126(6), 339–344. https://doi.org/10.1061/(ASCE)0733-9496(2000)126:6(339)

[ID=221] Lopez-Gunn, E. (2003). The Role of Collective Action in Water Governance: A Comparative Study of Groundwater User Associations in La Mancha Aquifers in Spain. *Water International*, 28(3), 367–378. https://doi.org/10.1080/02508060308691711

[ID=222] Lopez-Rodriguez, M. D., Castro, A. J., Castro, H., Jorreto, S., & Cabello, J. (2015). Science-policy interface for addressing environmental problems in arid Spain. *Environmental Science & Policy*, 50, 1–14. https://doi.org/10.1016/j.envsci.2015.01.013

[ID=223] Lubell, M., & Lippert, L. (2011). Integrated regional water management: a study of collaboration or water politics-as-usual in California, USA. *International Review of Administrative Sciences*, 77(1), 76–100. https://doi.org/10.1177/0020852310388367

[ID=225] Lubell, M. (2007). Familiarity breeds trust: Collective action in a policy domain. *Journal of Politics*, 69(1), 237–250. https://doi.org/10.1111/j.1468-2508.2007.00507.x

[ID=227] Madhava Chandran, K., Joseph, E. J., & Sushanth, C. M. (2016). Performance Evaluation of Selected Irrigation Systems in Kerala State, India. *Irrigation and Drainage*, 65(5), 613–619. https://doi.org/10.1002/ird.2012

[ID=228] Maharaj, V., & Pietersen, T. (2004). Consulting South Africa’s diverse population about the country’s proposed National Water Resource Strategy. *Water SA*, 30(5), 673–680. Retrieved from https://www.scopus.com/inward/record.uri?eid=2-s2.0-11944261291&partnerID=40&md5=e586b170c8806e3b945f63ec8722314e

[ID=229] Maheshwari, B., Varua, M., Ward, J., Packham, R., Chinnasamy, P., Dashora, Y., Rao, P. (2014). The role of transdisciplinary approach and community participation in village scale groundwater management: Insights from Gujarat and Rajasthan, India. *Water (Switzerland)*, 6(11), 3386–3408. https://doi.org/10.3390/w6113386

[ID=232] Máñez, M., Froebrich, J., Ferrand, N., & Silva, A. (2007). Participatory dam systems modelling: A case study of the transboundary Guadiana River in the Iberian Peninsula (B. L.J., Ed.). *Water Science and Technology*, Vol. 56, pp. 145–156. https://doi.org/10.2166/wst.2007.546

[ID=233] Mankad, A., & Walton, A. (2015). Accepting managed aquifer recharge of urban storm water reuse: The role of policy-related factors. *Water Resources Research*, 51(12), 9696–9707. https://doi.org/10.1002/2015WR017633

[ID=234] Marks, J. S., & Zadoroznyj, M. (2005). Managing sustainable urban water reuse: Structural context and cultures of trust. *Society and Natural Resources*, 18(6), 557–572. https://doi.org/10.1080/08941920590947995

[ID=235] Marks, J. S. (2004). Advancing community acceptance of reclaimed water. Water, 31(5), 46–51. Retrieved from https://www.scopus.com/inward/record.uri?eid=2-s2.0-31344461100&partnerID=40&md5=12dbdd896bde3ccf17dca4073c586668

[ID=236] Mase, A. S., Babin, N. L., Prokopy, L. S., & Genskow, K. D. (2015). Trust in Sources of Soil and Water Quality Information: Implications for Environmental Outreach and Education. *Journal of the American Water Resources Association*, 51(6), 1656–1666. https://doi.org/10.1111/1752-1688.12349

[ID=237] Mason, S. A. (2005). Are we scorpions? The role of upstream-downstream dialogue in fostering cooperation in the Nile Basin. *Mountain Research and Development*, 25(2), 115–120. https://doi.org/10.1659/0276-4741(2005)025[0115:AWS]2.0.CO;2

[ID=238] Massoud, M. A., Kazarian, A., Alameddine, I., & Al-Hindi, M. (2018). Factors influencing the reuse of reclaimed water as a management option to augment water supplies. *Environmental Monitoring and Assessment*, 190(9). https://doi.org/10.1007/s10661-018-6905-y

[ID=239] McCord, P., Waldman, K., Baldwin, E., Dell’Angelo, J., & Evans, T. (2018). Assessing multi-level drivers of adaptation to climate variability and water insecurity in smallholder irrigation systems. *World Development*, 108, 296–308. https://doi.org/10.1016/j.worlddev.2018.02.009

[ID=241] McIntyre-Mills, J., & Wirawan, R. (2018). Cascading risks of climate change on water security and the potential for rapid adapatation Consequences of modernity’, potential of the double hermeneutic and implications for human security. Conference Proceding. Retrieved from https://www.scopus.com/inward/record.uri?eid=2-s2.0-85064265897&partnerID=40&md5=054f7ea5e34f4861a7fc834f6207879b

[ID=242] McKay, J. M. (2011). Australian water allocation plans and the sustainability objective-conflicts and conflict-resolution measures . *Hydrological Sciences Journal*, 56(4), 615–629. https://doi.org/10.1080/02626667.2011.580456

[ID=243] McMillan, H. K., Westerberg, I. K., & Krueger, T. (2018). Hydrological data uncertainty and its implications. *Wiley Interdisciplinary Reviews-Water*, 5(6). https://doi.org/10.1002/wat2.1319

[ID=244] McQuigge, M. (2002). Water: A clear and present danger. *Canadian Journal of Public Health*, 93(1), 10–11. Retrieved from https://www.scopus.com/inward/record.uri?eid=2-s2.0-0036236377&partnerID=40&md5=66b6887316f326120bd7c7df3fd2b29f

[ID=245] Medema, W., Wals, A., & Adamowski, J. (2014). Multi-Loop Social Learning for Sustainable Land and Water Governance: Towards a Research Agenda on the Potential of Virtual Learning Platforms. *NJAS - Wageningen Journal of Life Sciences*, 69, 23–38. https://doi.org/10.1016/j.njas.2014.03.003

[ID=246] Meinzen-Dick, R., Janssen, M. A., Kandikuppa, S., Chaturvedi, R., Rao, K., & Theis, S. (2018). Playing games to save water: Collective action games for groundwater management in Andhra Pradesh, India. *World Development*, 107, 40–53. https://doi.org/10.1016/j.worlddev.2018.02.006

[ID=247] Merrey, D. J., Prakash, A., Swatuk, L., Jacobs, I., & Narain, V. (2017). Water Governance Futures in South Asia and Southern Africa: Déjà Vu All Over Again? *Global Issues in Water Policy*, Vol. 6, pp. 229–250. https://doi.org/10.1007/978-3-319-43350-9_12

[ID=248] Metawie, A. F. (2004). History of co-operation in the Nile basin. *International Journal of Water Resources Development*, 20(1), 47–63. https://doi.org/10.1080/07900620310001635601

[ID=249] Michetti, M., Raggi, M., Guerra, E., & Viaggi, D. (2019). Interpreting Farmers’ Perceptions of Risks and Benefits Concerning Wastewater Reuse for Irrigation: A Case Study in Emilia-Romagna (Italy). *WATER*, 11(1). https://doi.org/10.3390/w11010108

[ID=250] Minkman, E., Rutten, M. M., & van der Sanden, M. C. A. (2017). Acceptance of Mobile Technology for Citizen Science in Water Resource Management. *Journal of Irrigation and Drainage Engineering*, 143(3, SI). https://doi.org/10.1061/(ASCE)IR.1943-4774.0001043

[ID=251] Mirzaei, A., Knierim, A., Fealy Nahavand, S., Shokri, S. A., & Mahmoudi, H. (2019). Assessment of policy instruments towards improving the water reservoirs’ governance in Northern Iran. *Agricultural Water Management*, 211, 48–58. https://doi.org/10.1016/j.agwat.2018.09.020

[ID=252] Mitchell, M., Curtis, A., Sharp, E., & Mendham, E. (2012). Directions for social research to underpin improved groundwater management. *Journal of Hydrology*, 448–449, 223–231. https://doi.org/10.1016/j.jhydrol.2012.04.056

[ID=253] Molenaar, K. R., Bogus, S. M., & Priestley, J. M. (2004). Design/build for water/wastewater facilities: State of the industry survey and three case studies. *Journal of Management in Engineering*, 20(1), 16–24. https://doi.org/10.1061/(ASCE)0742-597X(2004)20:1(16)

[ID=255] Moody, M. (2009). Everyone will get better together: How those responsible for california’s bay-delta water system understand collaboration. *International Review of Public Administration*, 13, 13–32. https://doi.org/10.1080/12294659.2009.10805137

[ID=256] Moore, M.-L., Shaw, K., & Castleden, H. (2018). “‘We need more data!’” the politics of scientific information for water governance in the context of hydraulic fracturing. *Water Alternatives*, 11(1), 142–162. Retrieved from https://www.scopus.com/inward/record.uri?eid=2-s2.0-85041570056&partnerID=40&md5=39435f7464273cabc88fe3e4a985057a

[ID=258] Moshtagh, M., & Mohsenpour, M. (2018). Community viewpoints about water crisis, conservation and recycling: a case study in Tehran. Environment, *Development and Sustainability*, pp. 1–11. https://doi.org/10.1007/s10668-018-0158-3

[ID=259] Mott Lacroix, K. E., & Megdal, S. B. (2016). Explore, synthesize, and repeat: Unraveling complex water management issues through the stakeholder engagement wheel. *Water (Switzerland)*, 8(4). https://doi.org/10.3390/w8040118

[ID=260] Nagata, J. M., Valeggia, C. R., Smith, N. W., Barg, F. K., Guidera, M., & Bream, K. D. W. (2011). Criticisms of chlorination: Social determinants of drinking water beliefs and practices among the Tz’utujil Maya. *Revista Panamericana de Salud Publica/Pan American Journal of Public Health*, 29(1), 9–16. Retrieved from https://www.scopus.com/inward/record.uri?eid=2-s2.0-79952275659&partnerID=40&md5=1c6300e8f2d9e96f9290c94436617953

[ID=261] Nancarrow, B. E., Leviston, Z., & Tucker, D. I. (2009). Measuring the predictors of communities’ behavioural decisions for potable reuse of wastewater. *Water Science and Technology*, Vol. 60, pp. 3199–3209. https://doi.org/10.2166/wst.2009.759

[ID=262] Nancarrow, B. E., Leviston, Z., Po, M., Porter, N. B., & Tucker, D. I. (2008). What drives communities’ decisions and behaviours in the reuse of wastewater. *Water Science and Technology*, Vol. 57, pp. 485–491. https://doi.org/10.2166/wst.2008.160

[ID=264] Nickum, J. E. (2012). Exploring the Boundaries of Water Quality Management in Asia. International Journal of *Water Resources Development*, 28(2), 233–245. https://doi.org/10.1080/07900627.2012.668647

[ID=265] Noemdoe, S., Jonker, L., & Swatuk, L. A. (2006). Perceptions of water scarcity: The case of Genadendal and outstations. *Physics and Chemistry of the Earth*, 31(15–16), 771–778. https://doi.org/10.1016/j.pce.2006.08.003

[ID=266] Nykvist, B., Borgström, S., & Boyd, E. (2017). Assessing the adaptive capacity of multi-level water governance: ecosystem services under climate change in Mälardalen region, Sweden. *Regional Environmental Change*, 17(8), 2359–2371. https://doi.org/10.1007/s10113-017-1149-x

[ID=268] Olsson, J. A., & Andersson, L. (2007). Possibilities and problems with the use of models as a communication tool in water resource management. *Water Resources Management*, 21(1), 97–110. https://doi.org/10.1007/s11269-006-9043-1

[ID=269] Olsson, J. A., & Berg, K. (2005). Local stakeholders’ acceptance of model-generated data used as a communication tool in water management: The Rönneå study. *Ambio*, 34(7), 507–512. Retrieved from https://www.scopus.com/inward/record.uri?eid=2-s2.0-28544438571&partnerID=40&md5=bd70900d069d3b1378abc2f8074b6aa6

[ID=270] Omid, M. H., Akbari, M., Zarafshani, K., Eskandari, G. H., & Fami, H. S. (2012). Factors influencing the success of water user associations in Iran: A case of Moqan, Tajan, and Varamin. *Journal of Agricultural Science and Technology*, 14(1), 27–36. Retrieved from https://www.scopus.com/inward/record.uri?eid=2-s2.0-84975806517&partnerID=40&md5=b96f319b2abd4383be4fa80480493fc9

[ID=271] Onencan, A. M., Enserink, B., & Van de Walle, B. (2018). A study of trust and cooperation in the Nzoia river basin using a water policy game. *Sustainability (Switzerland)*, 10(12). https://doi.org/10.3390/su10124678

[ID=272] Ormerod, K. J., & Scott, C. A. (2013). Drinking Wastewater: Public Trust in Potable Reuse*. Science Technology and Human Values*, 38(3), 351–373. https://doi.org/10.1177/0162243912444736

[ID=273] Owen, G., & Goldin, J. (2015). Assessing the relationship between youth capabilities and food security: A case study of a rainwater harvesting project in South Africa. *Water SA*, 41(4), 541–548. https://doi.org/10.4314/wsa.v41i4.14

[ID=274] Paerregaard, K. (2013). Governing Water in the Andean Community of Cabanaconde, Peru From Resistance to Opposition and to Cooperation (and Back Again?). *Mountain Research and Development*, 33(3), 207–214. https://doi.org/10.1659/MRD-JOURNAL-D-12-00107.1

[ID=275] Panten, K., van Heel, B. F., Fliervoet, J. M., & van den Born, R. J. G. (2018). Cross-Border Collaboration in River Management: Views on Participation in a Dutch-German Case Study. *Water Resources Management*, 32(12), 4063–4078. https://doi.org/10.1007/s11269-018-2039-9

[ID=276] Parag, Y., & Timmons Roberts, J. (2009). A battle against the bottles: Building, claiming, and regaining tap-water trustworthiness. *Society and Natural Resources*, 22(7), 625–636. https://doi.org/10.1080/08941920802017248

[ID=277] Pareja, C., Honey-Rosés, J., Kunz, N. C., Fraser, J., & Xavier, A. (2018). What participation? Distinguishing water monitoring programs in mining regions based on community participation. *Water (Switzerland)*, 10(10). https://doi.org/10.3390/w10101325

[ID=278] Park, H., Tsusaka, T. W., Pede, V. O., & Kim, K.-M. (2017). The Impact of a Local Development Project on Social Capital: Evidence from the Bohol Irrigation Scheme in the Philippines. *WATER*, 9(3). https://doi.org/10.3390/w9030202

[ID=280] Patrick, R. J., Machial, L., Quinney, K., & Quinney, L. (2017). Lessons learned through community-engaged planning. *International Indigenous Policy Journal*, 8(2). https://doi.org/10.18584/iipj.2017.8.2.7

[ID=282] Pennington, J. H., Steele, M. A., Teague, K. A., Kurz, B., Gbur, E., Popp, J., … Nelson, M. A. (2008). Breaking ground: A cooperative approach to collecting information on conservation practices from an initially uncooperative population. *Journal of Soil and Water Conservation*, 63(6), 208A-211A. https://doi.org/10.2489/jswc.63.6.2o8A

[ID=283] Petty, T. R., Gongwer, J. B., & Schnabel, W. (2018). Bridging policy and science action boundaries: information influences on US congressional legislative key staff decision making in natural resources. *Policy Sciences*, 51(1), 77–96. https://doi.org/10.1007/s11077-018-9311-y

[ID=285] Phillips, C., Allen, W., Fenemor, A., Bowden, B., & Young, R. (2010). Integrated catchment management research: lessons for interdisciplinary science from the Motueka Catchment, New Zealand. *Marine and Freshwater Research*, 61(7), 749–763. https://doi.org/10.1071/MF09099

[ID=286] Phumpiu, P., & Gustafsson, J. E. (2009). When are partnerships a viable tool for development? Institutions and partnerships for water and sanitation service in Latin America. *Water Resources Management*, 23(1), 19–38. https://doi.org/10.1007/s11269-008-9262-8

[ID=289] Qian, N. (2018). Bottled water or tap water? A comparative study of drinking water choices on university campuses. *Water (Switzerland)*, 10(1). https://doi.org/10.3390/w10010059

[ID=291] Radatz, A. M., Herron, C. L., Cooley, E. T., Genskow, K., & Ruark, M. D. (2018). Improving water quality knowledge through a focus on partnership: A University of Wisconsin Discovery Farms case study. *Journal of Soil and Water Conservation*, 73(1), 16A-21A. https://doi.org/10.2489/jswc.73.1.16A

[ID=292] Raj, P. (2011). Groundwater resource assessment, categories, and typologies: case study, Andhra Pradesh, India. *Environmental Monitoring and Assessment*, 173(1–4), 777–788. https://doi.org/10.1007/s10661-010-1422-7

[ID=293] Renner, R., Schneider, F., Hohenwallner, D., Kopeinig, C., Kruse, S., Lienert, J., … Muhar, S. (2013). Meeting the challenges of transdisciplinary knowledge production for sustainable water governance. *Mountain Research and Development*, 33(3), 234–247. https://doi.org/10.1659/MRD-JOURNAL-D-13-00002.1

[ID=295] Rosario, S., Ronald, K. A., & Giardino, J. R. (2010). Water planning under an unsustainable condition: Challenges for the Laguna region, Mexico. Conference Paper. https://doi.org/10.1061/41114(371)225

[ID=296] Roßner, R., & Zikos, D. (2018). The Role of Homogeneity and Heterogeneity Among Resource Users on Water Governance: Lessons Learnt from an Economic Field Experiment on Irrigation in Uzbekistan. *Water Economics and Policy*, 4(3). https://doi.org/10.1142/S2382624X1850008X

[ID=297] Roth, D., & Winnubst, M. (2014). Moving out or living on a mound? Jointly planning a Dutch flood adaptation project. *Land Use Policy*, 41, 233–245. https://doi.org/10.1016/j.landusepol.2014.06.001

[ID=298] Rothenberger, D., Frei, U., & Brugger, F. (2005). Policy principles and implementation guidelines for private sector participation in the water sector - A step towards better results. *Water Science and Technology*, Vol. 51, pp. 61–69. Retrieved from https://www.scopus.com/inward/record.uri?eid=2-s2.0-21244505792&partnerID=40&md5=83a39f60584850e351fed9ced282cdda

[ID=299] Rouillard, J. J., & Spray, C. J. (2017). Working across scales in integrated catchment management: lessons learned for adaptive water governance from regional experiences. *Regional Environmental Change*, 17(7), 1869–1880. https://doi.org/10.1007/s10113-016-0988-1

[ID=302] Running, K., Burnham, M., & Du Bray V, M. (n.d.). Perceptions of fairness in common-pool resource access: farmer responses to new agricultural water use restrictions in Idaho. *Environmental Sociology*. https://doi.org/10.1080/23251042.2019.1643548

[ID=303] Ryder, D. S., Tomlinson, M., Gawne, B., & Likens, G. E. (2010). Defining and using best available science: A policy conundrum for the management of aquatic ecosystems. *Marine and Freshwater Research*, 61(7), 821–828. https://doi.org/10.1071/MF10113

[ID=304] Saarikoski, H., Primmer, E., Saarela, S.-R., Antunes, P., Aszalós, R., Baró, F., … Young, J. (2018). Institutional challenges in putting ecosystem service knowledge in practice. *Ecosystem Services*, 29, 579–598. https://doi.org/10.1016/j.ecoser.2017.07.019

[ID=305] Salamon, S., Farnsworth, R. L., & Rendziak, J. A. (1998). Is locally led conservation planning working? A farm town case study. *Rural Sociology*, 63(2), 214–234. https://doi.org/10.1111/j.1549-0831.1998.tb00672.x

[ID=306] Sally, H., Lévite, H., & Cour, J. (2011). Local water management of small reservoirs: Lessons from two case studies in Burkina Faso. *Water Alternatives*, 4(3), 365–382. Retrieved from https://www.scopus.com/inward/record.uri?eid=2-s2.0-83755178859&partnerID=40&md5=73a803f8a2fc9ba929b6e54d6218ef16

[ID=307] Sanbongi, K. (1991). New legal viewpoints for development and conservation of enclosed coastal seas. *Marine Pollution Bulletin*, 23(C), 563–566. Retrieved from https://www.scopus.com/inward/record.uri?eid=2-s2.0-0025854714&partnerID=40&md5=bf4852bba801befe1f9d84e478e82ceb

[ID=308] Satein, H., & Weber, E. (2018). Fighting to Cooperate: Litigation, Collaboration, and Water Management in the Upper Deschutes River Basin, Oregon. *Case Studies in the Environment*, 2(1). https://doi.org/10.1525/cse.2018.001115

[ID=309] Schneider, F., & Rist, S. (2014). Envisioning sustainable water futures in a transdisciplinary learning process: combining normative, explorative, and participatory scenario approaches. *Sustainability Science*, 9(4), 463–481. https://doi.org/10.1007/s11625-013-0232-6

[ID=312] Seher, W., & Löschner, L. (2018). Balancing upstream–downstream interests in flood risk management: experiences from a catchment-based approach in Austria. *Journal of Flood Risk Management*, 11(1), 56–65. https://doi.org/10.1111/jfr3.12266

[ID=313] Shamsuzzoha, M., Kormoker, T., & Ghosh, R. C. (2018). Implementation of Water Safety Plan Considering Climatic Disaster Risk Reduction in Bangladesh: A Study on Patuakhali Pourashava Water Supply System (A. D. & H. R., Eds.). https://doi.org/10.1016/j.proeng.2018.01.075

[ID=314] Sheer, A. M. S. (2014). We C.A.N. do it: Actively engaging stakeholders in modelling. *Conference Proceedings.* Retrieved from https://www.scopus.com/inward/record.uri?eid=2-s2.0-84911865424&partnerID=40&md5=001605e3ece84ac7deac8b4df477218d

[ID=316] Siegmund-Schultze, M., Rodorff, V., Köppel, J., & Sobral, M. D. C. (2015). Paternalism or participatory governance? Efforts and obstacles in implementing the Brazilian water policy in a large watershed. *Land Use Policy*, 48, 120–130. https://doi.org/10.1016/j.landusepol.2015.05.024

[ID=317] Siew, T. F., Aenis, T., Spangenberg, J. H., Nauditt, A., Döll, P., Frank, S. K., Wang, J. (2016). Transdisciplinary research in support of land and water management in China and Southeast Asia: evaluation of four research projects. *Sustainability Science*, 11(5), 813–829. https://doi.org/10.1007/s11625-016-0378-0

[ID=318] Siew, T. F., Döll, P., & Yimit, H. (2014). Experiences with a transdisciplinary research approach for integrating ecosystem services into water management in Northwest China. In The Global Water System in the Anthropocene: Challenges for Science and Governance (pp. 303–319). https://doi.org/10.1007/978-3-319-07548-8_20

[ID=319] Sixt, G. N., Klerkx, L., & Griffin, T. S. (2018). Transitions in water harvesting practices in Jordan’s rainfed agricultural systems: Systemic problems and blocking mechanisms in an emerging technological innovation system. *Environmental Science and Policy*, 84, 235–249. https://doi.org/10.1016/j.envsci.2017.08.010

[ID=320] Skarbøvik, E., Shumka, S., Mukaetov, D., & Nagothu, U. S. (2010). Harmonised monitoring of Lake Macro Prespa as a basis for integrated water resources management. *Irrigation and Drainage Systems*, 24(3–4), 223–238. https://doi.org/10.1007/s10795-010-9099-1

[ID=321] Slomp, R., Kolen, B., Westera, H., Verweij, J., & Riedstra, D. (2016). Interpreting the impact of flood forecasts by combining policy analysis studies and flood defence. Conference Proceedings https://doi.org/10.1051/e3sconf/20160703006

[ID=322] Ssozi-Mugarura, F., Blake, E., & Rivett, U. (2017). Codesigning with communities to support rural water management in Uganda. *CoDesign*, 13(2), 110–126. https://doi.org/10.1080/15710882.2017.1310904

[ID=324] Stewart, J., & Bennett, M. (2017). Integrated watershed management in the Bow River basin, Alberta: experiences, challenges, and lessons learned. *International Journal of Water Resources Development*, 33(3), 458–472. https://doi.org/10.1080/07900627.2016.1238345

[ID=325] Sullivan, A., White, D. D., & Hanemann, M. (2019). Designing collaborative governance: Insights from the drought contingency planning process for the lower Colorado River basin. *Environmental Science and Policy*, 91, 39–49. https://doi.org/10.1016/j.envsci.2018.10.011

[ID=328] Tegoni, C., Mulazzani, L., & Setti, M. (2016). Water governance under uncertainty: The case study of Users’ Associations in Lebanon. *New Medit*, 15(1), 62–71. Retrieved from https://www.scopus.com/inward/record.uri?eid=2-s2.0-84991449227&partnerID=40&md5=e2e302810bd7dd35a8745c9b13ad4940

[ID=330] Theesfeld, I. (2004). Constraints on Collective Action in a Transitional Economy: The Case of Bulgaria’s Irrigation Sector. *World Development*, 32(2), 251–271. https://doi.org/10.1016/j.worlddev.2003.11.001

[ID=331] Tkachenko, N., Procter, R., & Jarvis, S. (2016). From kontroll verlust to kontroll gewinn: Rediscovering a Meso-level normative hierarchy for integrated water management in England using on-line activities. WS-16-16-WS-16-20, 199–202. Retrieved from https://www.scopus.com/inward/record.uri?eid=2-s2.0-85021878218&partnerID=40&md5=4fc54dd61ba6aa51f0c1331fad5bc686

[ID=332] Tromp, E., & Bots, P. (2018). Enhancing local knowledge uptake through stakeholder engagement in Dutch dike redesign. Conference Proceeding. Retrieved from https://www.scopus.com/inward/record.uri?eid=2-s2.0-85055505539&partnerID=40&md5=75d5b972be4d1f81c5ab55d5e65eeef3

[ID=333] Totin, E., van Mierlo, B., Saidou, A., Mongbo, R., Agbossou, E., Stroosnijder, L., & Leeuwis, C. (2012). Barriers and opportunities for innovation in rice production in the inland valleys of Benin. NJAS-Wageningen Journal of Life Sciences, 60–63(SI), 57–66. https://doi.org/10.1016/j.njas.2012.06.001

[ID=335] Ulibarri, N., Cain, B. E., & Ajami, N. K. (2017). A Framework for Building Efficient Environmental Permitting Processes. *SUSTAINABILITY*, 9(2). https://doi.org/10.3390/su9020180

[ID=336] Ulibarri, N. (2018). Collaborative model development increases trust in and use of scientific information in environmental decision-making. *Environmental Science and Policy*, 82, 136–142. https://doi.org/10.1016/j.envsci.2018.01.022

[ID=337] Vái, A., Ereifej, L., & Ferencz, Z. (2009). Implementing the EU water framework directive in hungary: A pilot project in the upper-tisza region. *International Journal of Risk Assessment and Management*, 12(1), 82–102. https://doi.org/10.1504/IJRAM.2009.024131

[ID=338] Van De Meene, S. J., Brown, R. R., & Farrelly, M. A. (2010). Capacity attributes of future urban water management regimes: Projections from Australian sustainability practitioners. *Water Science and Technology*, 61(9), 2241–2250. https://doi.org/10.2166/wst.2010.154

[ID=339] van Popering-Verkerk, J., & van Buuren, A. (2017). Developing collaborative capacity in pilot projects: Lessons from three Dutch flood risk management experiments. *Journal of Cleaner Productions*, 169, 225–233. https://doi.org/10.1016/j.jclepro.2017.04.141

[ID=340] van Vliet, B., & Stein, N. (2004). New consumer roles in waste water management. *Local Environment*, 9(4), 353–366. https://doi.org/10.1080/1354983042000246270

[ID=341] van Woerkum, C. (2007). Raising awareness on water and climate related risks - An overview. *Water Science and Technology*, Vol. 56, pp. 63–70. https://doi.org/10.2166/wst.2007.537

[ID=342] Varady, R. G., Zuniga-Teran, A. A., Gerlak, A. K., & Megdal, S. B. (2016). Modes and approaches of groundwater governance: A survey of lessons learned from selected cases across the globe. *Water (Switzerland)*, 8(10). https://doi.org/10.3390/w8100417

[ID=343] Vári, A. (2004). Hungarian experiences with public participation in water management. *Water International*, 29(3), 329–337. https://doi.org/10.1080/02508060408691787

[ID=344] Varua, M. E., Ward, J., Maheshwari, B., Oza, S., Purohit, R., Hakimuddin, & Chinnasamy, P. (2016). Assisting community management of groundwater: Irrigator attitudes in two watersheds in Rajasthan and Gujarat, India. *Journal of Hydrology*, 537, 171–186. https://doi.org/10.1016/j.jhydrol.2016.02.003

[ID=345] Vedachalam, S., & Mancl, K. M. (2010). Water resources and wastewater reuse: Perceptions of students at the ohio state university campus. *Ohio Journal of Science*, 110(5), 104–113. Retrieved from https://www.scopus.com/inward/record.uri?eid=2-s2.0-84863432409&partnerID=40&md5=5f26a503bed1159c6389a94e762d3de3

[ID=346] Verbrugge, L. N. H., Ganzevoort, W., Fliervoet, J. M., Panten, K., & van den Born, R. J. G. (2017). Implementing participatory monitoring in river management: The role of stakeholders’ perspectives and incentives. *Journal of Environmental Management*, 195, 62–69. https://doi.org/10.1016/j.jenvman.2016.11.035

[ID=347] Vertessy, R. (2013). Water information services for Australians. *Australian Journal of Water Resources*, 16(2), 91–106. https://doi.org/10.7158/W13-MO01.2013.16.2

[ID=348] Villamayor-Tomas, S., Thiel, A., Villamayor-Tomas, S., Fleischman, F. D., Ibarra, I. P., & van Laerhoven, F. (2014). From Sandoz to Salmon: Conceptualizing resource and institutional dynamics in the Rhine watershed through the SES framework. *International Journal of the Commons*, 8(2), 361–395. https://doi.org/10.18352/ijc.411

[ID=349] Vinke-De Kruijf, J., Augustijn, D. C. M., & Bressers, H. T. A. (2012). Evaluation of Policy Transfer Interventions: Lessons from a Dutch-Romanian Planning Project. *Journal of Environmental Policy & Planning*, 14(2), 139–160. https://doi.org/10.1080/1523908X.2012.680700

[ID=350] Wachinger, G., Keilholz, P., & O’Brian, C. (2018). The Difficult Path from Perception to Precautionary Action—Participatory Modeling as a Practical Tool to Overcome the Risk Perception Paradox in Flood Preparedness. *International Journal of Disaster Risk Science*, 9(4), 472–485. https://doi.org/10.1007/s13753-018-0203-8

[ID=351] Wagner, M., Kaiser, R., Kreuter, U., & Wilkins, N. (2007). Managing the commons Texas style: Wildlife management and ground-water associations on private lands. *Journal of the American Water Resources Association*, 43(3), 698–711. https://doi.org/10.1111/j.1752-1688.2007.00056.x

[ID=352] Walsh, C. J., Fletcher, T. D., Bos, D. G., & Imberger, S. J. (2015). Restoring a stream through retention of urban stormwater runoff: a catchment-scale experiment in a social-ecological system. *Freshwater Science*, 34(3), 1161–1168. https://doi.org/10.1086/682422

[ID=353] Wang, L., Zhang, L., Lv, J., Zhang, Y., & Ye, B. (2018). Public awareness of drinking water safety and contamination accidents: A case study in Hainan Province, China. *Water (Switzerland)*, 10(4). https://doi.org/10.3390/w10040446

[ID=354] Wang, Y., & Ching, L. (2013). Institutional legitimacy: An exegesis of normative incentives. *International Journal of Water Resources Development*, 29(4), 514–525. https://doi.org/10.1080/07900627.2013.787831

[ID=355] Ward, S., Abdelmeguid, H., Farmani, R., Memon, F. A., & Butler, D. (2011). Sustainable water management - Modelling acceptability for decision support: A methodology. Conference Proceeding. Retrieved from https://www.scopus.com/inward/record.uri?eid=2-s2.0-84906229346&partnerID=40&md5=8ced6cad20ff435629422e5ce8c210ab

[ID=357] Waterton, C., Maberly, S. C., Tsouvalis, J., Watson, N., Winfield, I. J., & Norton, L. R. (2015). Committing to Place: The Potential of Open Collaborations for Trusted Environmental Governance. *PLoS Biology*, 13(3). https://doi.org/10.1371/journal.pbio.1002081

[ID=359] Watts, B., & Everard, M. (2018). Economics of wetland conservation case study: Catchment management for water quality. In The Wetland Book: I: Structure and Function, Management, and Methods (pp. 925–929). https://doi.org/10.1007/978-90-481-9659-3_172

[ID=361] Weible, C. M. (2007). Stakeholder perceptions of scientists: Lake Tahoe environmental policy from 1984 to 2001. *Environmental Management*, 40(6), 853–865. https://doi.org/10.1007/s00267-007-9005-2

[ID=363] Westling, E. L., Sharp, L., Rychlewski, M., & Carrozza, C. (2014). Developing adaptive capacity through reflexivity: lessons from collaborative research with a UK water utility. *Critical Policy Studies*, 8(4), 427–446. https://doi.org/10.1080/19460171.2014.957334

[ID=364] Whaley, L., & Weatherhead, E. K. (2015). Using the politicized institutional analysis and development framework to analyze (Adaptive) comanagement: Farming and water resources in England. *Ecology and Society*, 20(3). https://doi.org/10.5751/ES-07769-200343

[ID=365] Whaley, L., & Weatherhead, E. K. (2015). Competition, conflict, and compromise: Three discourses used by irrigators in england and their implications for the co-management of water resources. *Water Alternatives*, 8(1), 800–819. Retrieved from https://www.scopus.com/inward/record.uri?eid=2-s2.0-84922310983&partnerID=40&md5=13241822d5d6bd8df55be0e29be7892f

[ID=366] Wheeler, S. A., Hatton MacDonald, D., & Boxall, P. (2017). Water policy debate in Australia: Understanding the tenets of stakeholders’ social trust. *Land Use Policy*, 63, 246–254. https://doi.org/10.1016/j.landusepol.2017.01.035

[ID=367] White, D. D., Rauh, E. K., Sullivan, A., Larson, K. L., Wutich, A., Linthicum, D., … Lawless, K. L. (2019). Public attitudes toward urban water sustainability transitions: a multi-city survey in the western United States. *Sustainability Science*. 14(6), 1469-1483. https://doi.org/10.1007/s11625-019-00658-z

[ID=368] Whitman, G. P., & Milledge, D. G. (2015). Going with the flow? Using participatory action research in physical geography. *Progress in Physical Geography*, 39(5), 622–639. https://doi.org/10.1177/0309133315589707

[ID=369] Whittington, D. (2016). Ancient Instincts: Implications for Water Policy in the 21st Century. *Water Economics and Policy*, 2(2). https://doi.org/10.1142/S2382624X16710028

[ID=370] Wilson, Z., Malakoana, M., & Gounden, T. (2008). Trusting consumers: Involving communities in municipal water service decision making in Durban. *Water SA*, 34(2), 141–146. Retrieved from https://www.scopus.com/inward/record.uri?eid=2-s2.0-47249130752&partnerID=40&md5=d1a9084a9419a3f90edac1508acf0f6f

[ID=371] Woelfle-Erskine, C. (2015). Rain tanks, springs, and broken pipes as emerging water commons along Salmon Creek, CA, USA. *ACME*, 14(3), 735–750. Retrieved from https://www.scopus.com/inward/record.uri?eid=2-s2.0-84945131211&partnerID=40&md5=60cc1cf800d1c6ba3d8b07912d38af0d

[ID=373] Wong, S. S., & Yelderman Jr., J. C. (2016). Time not wasted: How collaborative research and education help build groundwater sustainability in rural northern Uganda, Africa (G. J.K. & W. G.R., Eds.). Special Paper of the Geological Society of America, Vol. 520, pp. 183–192. https://doi.org/10.1130/2016.2520(17)

[ID=376] Xie, L., Rahaman, M. M., & Shen, W. (2018). When do institutions work? A comparison of two water disputes over the ganges, brahmaputra and meghna river basins. *Water Policy*, 20(2), 308–322. https://doi.org/10.2166/wp.2017.149

[ID=377] Xie, L., Zhang, Y., & Panda, J. P. (2018). Mismatched Diplomacy: China–India Water Relations Over the Ganges–Brahmaputra–Meghna River Basin. Journal of Contemporary China, 27(109), 32–46. https://doi.org/10.1080/10670564.2017.1363014

[ID=378] Yang, L., Rezitis, A., Zhu, Y., & Ren, Y. (2018). Investigating the Effects of Social Trust and Perceived Organizational Support on Irrigation Management Performance in Rural China. *WATER*, 10(9). https://doi.org/10.3390/w10091252

[ID=379] Yoder, J., Roberts, V., Craun, G. F., Hill, V., Hicks, L. A., Alexander, N. T., … Roy, S. L. (2008). Surveillance for waterborne disease and outbreaks associated with drinking water and water not intended for drinking--United States, 2005-2006. MMWR. Surveillance Summaries : Morbidity and Mortality Weekly Report. *Surveillance Summaries / CDC*, 57(9), 39–62. Retrieved from https://www.scopus.com/inward/record.uri?eid=2-s2.0-52049121325&partnerID=40&md5=3c706a16f3b85ca3a3a6a70fc7f518f9

[ID=380] Yu, H., Edmunds, M., Lora-Wainwright, A., & Thomas, D. (2014). From principles to localized implementation: villagers’ experiences of IWRM in the Shiyang River basin, Northwest China. *International Journal of Water Resources Development*, 30(3), 588–604. https://doi.org/10.1080/07900627.2014.917949

[ID=381] Yue, C., Wang, J., Watkins, E., Bonos, S., Nelson, K., Murphy, J. A., … Horgan, B. (2017). Consumer preferences for information sources of turfgrass products and lawn care. *Agronomy Journal*, 109(4), 1726–1733. https://doi.org/10.2134/agronj2016.05.0310

[ID=382] Zaji, A. H., Bonakdari, H., & Gharabaghi, B. (2019). Applying upstream satellite signals and a 2-d error minimization algorithm to advance early warning and management of flood water levels and river discharge. *IEEE Transactions on Geoscience and Remote Sensing*, 57(2), 902–910. https://doi.org/10.1109/TGRS.2018.2862640

[ID=384] Zhang, L., Zhu, X., Heerink, N., & Shi, X. (2014). Does output market development affect irrigation water institutions? Insights from a case study in northern China. *Agricultural Water Management*, 131, 70–78. https://doi.org/10.1016/j.agwat.2013.09.008

[ID=385] Zhang, L., Hu, J., Li, Y., & Pradhan, N. S. (2018). Public-private partnership in enhancing farmers’ adaptation to drought: Insights from the Lujiang Flatland in the Nu River (Upper Salween) valley, China. *Land Use Policy*, 71, 138–145. https://doi.org/10.1016/j.landusepol.2017.11.034

[ID=386] Zwarteveen, M., Roth, D., & Boelens, R. (2005). Water rights and legal pluralism: Beyond analysis and recognition. In Liquid Relations: Contested Water Rights and Legal Complexity (pp. 254–268). Retrieved from https://www.scopus.com/inward/record.uri?eid=2-s2.0-31744438622&partnerID=40&md5=4153d5eedc92d1f0a78d0687108a479c

**Appendix C**: **Full Tables & Extra Tables**

**Table C1**: Issues of water governance

| Issues of water governance:  (Multiple answers allowed) | (N=308 articles)  % (n) | (N=200 articles)  % (n) | (N=92 articles)  % (n) |
| --- | --- | --- | --- |
| Water distribution | 33% (102) | 30% (60) | 32.5% (30) |
| Water quality | 30.5% (94) | 29.5% (59) | 35% (32) |
| Environmental conservation | 16.5% (51) | 15.5% (31) | 14% (13) |
| Flood management | 11.5% (35) | 12% (24) | 12% (11) |
| Drought management | 9.5% (29) | 10.5% (21) | 10% (9) |
| Other water issues | 46% (142) | 47.5% (95) | 43.5% (40) |
| Number of issues addressed:  (Single answer) | (N=308 articles)  % (n) | (N=200 articles)  % (n) | (N=92 articles)  % (n) |
| - A single issue | 70% (216) | 70%(140) | 66% (61) |
| - Two issues | 19% (59) | 20.5% (41) | 25% (23) |
| - More than two | 11% (33) | 9.5% (19) | 9% (8) |
| Total | 100% (308) | 100% (200) | 100% (92) |

**Table C2**: Geographic locations

| Geographic Location:  (Single answer) | (N=200 articles)  % (n) | Average number of citations of articles from subgroup (n) | (N=92 articles)  % (n) |
| --- | --- | --- | --- |
| Europe | 22% (44) | 18.6 | 30.5%(28) |
| North America (Canada-US-Mexico) | 21.5% (43) | 18.6 | 19.5%(18) |
| Asia | 18.5% (37) | 8.3 | 14%(13) |
| Oceania (Australia-NZ-Solomon) | 12% (24) | 20.6 | 13%(12) |
| Africa | 8% (16) | 8 | 9%(8) |
| Central & South America | 6.5% (13) | 15.5 | 5.5%(5) |
| Multiple Continents | 8.5% (17) | 19.2 | 7.5%(7) |
| None | 3% (6) | 51.6 | 1%(1) |
| Total | 100% (200) |  | 100%(92) |

**Table C3**: Geographic scale

| Geographic scale of investigation:  (Single answer) | (N=200 articles)  % (n) | (N=92 articles)  % (n) |
| --- | --- | --- |
| Region/watershed | 45.5% (91) | 53% (49) |
| Local, community, village, neighborhood | 15.5% (31) | 17.5% (16) |
| National | 12% (24) | 13% (12) |
| Cross-border/international | 8.5% (17) | 3% (3) |
| Comparative: Regional issues from different countries | 7.5% (15) | 4.5% (4) |
| Comparative: Local issues from different countries | 5.5% (11) | 7.5% (7) |
| Other (specified in text) | 1% (2) |  |
| Not Applicable | 4.5% (9) | 1% (1) |
| Total | 100% (200) | 100% (92) |

**Table C4**: Subjects & Objects of Trust

| **Subject of Trust (Truster)**  (multiple answers allowed) | % of articles in which this subject is mentioned (N=200 articles) |  | **Object of Trust (Trustee)**  (multiple answers allowed) | % of articles in which this object is mentioned (N=200 articles) |
| --- | --- | --- | --- | --- |
| 1) Individuals: |  |  | 1) Individuals: | 22% (44) |
| A) Ordinary citizens | 49% (97) |  | 2) Social groups:  (minority/indigenous/religious groups) | 16% (31) |
| B) Farmers | 26% (52) |  | 3) Private companies/firms: | 16% (32) |
| C) Environmentalists | 8% (16) |  | 4) NGO’s: | 20% (40) |
| D Government employees/Civil servants | 16% (32) |  | 5) Governmental organizations: |  |
| E) Water managers | 17% (33) |  | A) Regional and local public bodies responsible for water management? | 57% (113) |
| F) ‘Other’ individuals | 7% (14) |  | B) National agencies responsible for water management? | 34% (67) |
| 2) Social groups: |  |  | C) National/Federal Governments | 33% (65) |
| A) Farmer organizations | 10% (20) |  | D) Supranational governments (EU, UN, NATO) | 3% (6) |
| B) Environmental groups | 9% (18) |  | 6) Trust in formal institutions or rules: (i.e. legislation and norms regulating water use and management) |  |
| C) Religious groups | 1% (1) |  | A) Operating permits, municipal laws…. | 5% (10) |
| D) Minorities | 3% (5) |  | B) National level (e.g. Swedish Environmental Code) | 5% (10) |
| E) Indigenous groups | 6% (12) |  | C) Supranational /EU level (e.g. the EU Water Framework Directive) | 3% (5) |
| F) Other | 10% (20) |  | 7A) Trust in water related knowledge: | 20% (39) |
| 3) Private companies/firms: | 13% (26) |  | 7B) Trust in scientists: | 5% (9) |
| 4) NGO’s: | 13% (26) |  | 8) ‘Other’: | 12% (24) |
| 5) Governmental organizations: | 26% (51) |  |  |  |
| 6) Nation States | 11% (22) |  |  |  |
| 7) ‘Other’ | 24% (48) |  |  |  |
| Number of times “other’ is used to indicate a term designating multiple subjects of trust | 14% (28) |  | Number of times “other’ is used to indicate a term designating multiple objects of trust | 15% (29) |
| Total number of articles with various subjects of trust | 50% (99) |  | Total number of articles with various objects of trust | 59% (117) |

**Table C5**: Trust relations

| What type of relationships are studied?  (Multiple answers allowed) | Articles in which this relationship is mentioned  % (N=200) |
| --- | --- |
| 1. Trust of individual citizens in other individual-level actors (inter-personal trust) | 20% (39) |
| 1. Trust of individual citizens in non-state affiliated groups | 22% (43) |
| 1. Mutual trust relations between different non-state affiliated groups | 15% (29) |
| 1. Trust of individual citizens in government organizations (Institutional trust) | 55% (109) |
| 1. Mutual trust relations between non-state affiliated groups and government organizations | 28% (56) |
| 1. Mutual trust relations between different government organizations | 12% (24) |
| 7) Trust relations between Nation States | 6%(12) |

**Table C6**: The role played by trust in empirical analyses

| What type of (directional) claims do the empirical articles that involve trust make about the role played by trust? | (Total N=92)  % (n) |
| --- | --- |
| Trust Outcome | 18.5% (17) |
| Trust Explanatory | 52% (48) |
| Trust Outcome and Explanatory variable | 15% (14) |
| Trust is mediator/moderator/intermediate variable | 10% (9) |
| Not directional: Only level of trust assessed | 4.5% (4) |
| Total | 100% (92) |

**Table C7**: Sub-issues of water governance studies by the geographic locations of those studies

| Geographic Location | % of all articles (N=200) | How often are articles from this geographic subgroup cited  (on average) | % of all articles on ‘Flooding’ by geography | % of all articles on ‘Droughts’ by geography | % of all articles on ‘Water Quality’ by geography | % of all articles on ‘Water distribution’ by geography | % of all articles on ‘Conservation’ by geography |
| --- | --- | --- | --- | --- | --- | --- | --- |
| Europe | 22% (44) | 18.6 | 50% | 4.7% | 11.9% | 13.3% | 29% |
| North America (Canada-US-Mexico) | 21.5% (43) | 18.6 | 16.6% | 23.8% | 27.1% | 16.7% | 25.8% |
| Asia | 18.5% (37) | 8.3 | 12.5% | 19% | 15.2% | 23.3% | 6.4% |
| Oceania (Australia-NZ-Solomon) | 12% (24) | 20.6 | 4.2% | 23.8% | 11.9% | 8.3% | 22.6% |
| Africa | 8% (16) | 8 | 0% | 14.3% | 8.5% | 20% | 0% |
| Central & South America | 6.5% (13) | 15.5 | 4.2% | 4.7% | 8.5% | 8.3% | 6.7% |
| Multiple Continents | 8.5% (17) | 19.2 | 12.5% | 9.5% | 13.6% | 8.3% | 6.4% |
| None | 3% (6) | 51.3 | 0% | 0% | 3.4% | 1.7% | 0% |
| Total | 100% (200) |  | 100% (24) | 100%(21) | 100% (59) | 100% (60) | 100% (31) |

**Table C8**: Conceptualization of trust by water governance sub-issues (using N=200 articles)

| Sub-issues (n=number of articles in group)  (Multiple entries allowed) | Sub-issue subgroup average trust count score | % of articles that defined trust within this sub-issue % Yes (n) | % of articles that distinguishes subtypes of trust within this sub-issue  % Yes (n) | % of articles that adopts research question on trust within this sub-issue  % Yes (n) |
| --- | --- | --- | --- | --- |
| Flood management (24) | 21.6 | 13% (3) | 8% (2) | 13% (3) |
| Drought management (21) | 32.6 | 5% (1) | 5% (1) | 14% (3) |
| Water quality (59) | 33.5 | 7% (4) | 17% (10) | 22% (13) |
| Water distribution (60) | 31.8 | 12% (7) | 15% (9) | 20% (12) |
| Environmental Conservation (31) | 19.1 | 10% (3) | 13% (4) | 17% (5) |
| Other water issues (95) | 28.1 | 17% (16) | 21% (20) | 16% (15) |

**Table C9**: Conceptualization by geographic location of studies (using N=200 articles)

| Geographic Location (n=number of articles in group) | By geographic location subgroup: average trust count | % of articles that defined trust within this location % Yes (n) | % of articles that distinguishes subtypes of trust within this location  % Yes (n) | % of articles that adopts research question on trust within this location  % Yes (n) |
| --- | --- | --- | --- | --- |
| Europe (44) | 25.3 | 23% (10) | 20% (9) | 14% (6) |
| North America (Canada-US-Mexico) (43) | 24.6 | 7% (3) | 16% (7) | 14% (6) |
| Asia (37) | 17.9 | 3% (1) | 8% (3) | 8% (3) |
| Oceania (Australia-NZ-Solomon) (24) | 24.6 | 13% (3) | 8% (2) | 25% (6) |
| Africa (16) | 56.4 | 19% (3) | 25% (4) | 25% (4) |
| Central & South America (13) | 21.7 | 8% (1) | 8% (1) | 33% (4) |
| Multiple Continents (17) | 23.1 | 12% (2) | 24% (4) | 29% (5) |
| None (6) | 32.7 | 0 % (0) | 33% (2) | 0% (0) |
| Total (N=200) |  |  |  |  |

**Table C10**: Conceptualization by geographic scale of studies

| Geographic Scale (n=number of articles in group) | By geographic scale: average trust count | % of articles that defined trust within this scale  % Yes (n) | % of articles that distinguishes subtypes of trust within this scale  % Yes (n) | % of articles that adopts research question on trust within this scale  % Yes (n) |
| --- | --- | --- | --- | --- |
| A single region or watershed (single country) (91) | 28.5 | 13% (12) | 14% (13) | 16% (15) |
| Local, community, village, neighborhood (single country) (31) | 23.3 | 13% (4) | 13% (4) | 23% (7) |
| National level (single country) (24) | 29.1 | 13% (3) | 25% (6) | 21% (5) |
| Cross-border/international (17) | 14.5 | 6% (1) | 6% (1) | 0% (0) |
| Comparative: Regional issues from different countries (15) | 18.1 | 7% (1) | 13% (2) | 13.3% (2) |
| Comparative: Local issues from different countries (11) | 30.4 | 9% (1) | 27% (3) | 45% (5) |
| Other (specified in text) (2) | 13.5 | 0% (0) | 0% (0) | 0% (0) |
| Not Applicable (9) | 33.3 | 11% (1) | 33.3% (3) | 0% (0) |
| Total (N=200) |  |  |  |  |
